# Regulation of NAT1 activity in modern humans by a novel phosphorylation site

**DOI:** 10.1126/sciadv.ady1666

**Published:** 2026-06-05

**Authors:** Luise Fast, Mikel Lana Alberro, Eva Rakava, Richard Ågren, Stephan Riesenberg, Wieland B. Huttner, Janet Kelso, Svante Pääbo, Hugo Zeberg

**Affiliations:** ^1^ Max Planck Institute for Evolutionary Anthropology, Leipzig, Germany.; ^2^ Karolinska Institutet, Stockholm, Sweden.; ^3^ Max Planck Institute of Molecular Cell Biology and Genetics, Dresden, Germany.; ^4^ Okinawa Institute of Science and Technology, Onna-son, Japan.

## Abstract

*N*-acetyltransferase 1 (NAT1) is an enzyme that acetylates certain drugs and carcinogens and is involved in folate metabolism. Some NAT1 variants are associated with susceptibility to cancer and birth defects. We show that while nearly all present-day humans carry a valine residue at position 149 and a serine residue at position 214, some carry the ancestral amino acids isoleucine and alanine at these positions because of gene flow from Neanderthals. Neither substitution affects enzymatic activity. However, replacing the serine residue, a known phosphorylation site, with a phosphomimetic aspartate reduces NAT1 activity by ~60%. Phosphorylation at this site may allow NAT1 to be down-regulated during pregnancy to reduce birth defect risk, while maintaining high activity in adults for xenobiotic clearance. This regulatory site is a molecular feature that differs between modern humans and Neanderthals.

## Teaser

NAT1 activity may be regulated by phosphorylation in modern humans but not in Neanderthals.

## INTRODUCTION

*N*-acetyltransferase 1 (NAT1) is an enzyme that transfers acetyl groups from the cofactor acetyl–coenzyme A to various drugs and carcinogens (*1*). NAT1 also plays a crucial role in folate clearance (*2*). Genetic variants of NAT1 have been linked to susceptibility to cancer (*3*) and birth defects such as spina bifida (*4*, *5*). A previous study identified a divergent *NAT1* haplotype found exclusively outside Africa (*6*). This haplotype, known as NAT1*11, carries two missense variants compared to the human reference sequence (rs4987076 Val149Ile and rs4986783 Ser214Ala). Several studies have investigated the functional effects of NAT1*11 in different experimental systems, yielding conflicting results (table S1). However, recent annotations classify this haplotype as encoding enzymes with increased activity compared to the common NAT1 enzyme in humans (*7*).

Here, we investigate the evolutionary origins and functional significance of NAT1*11 by studying its presence in Neanderthals and modern humans. We perform a detailed analysis of the individual amino acids’ contributions to NAT1 enzyme activity, identify possible regulatory differences between the Neanderthal and modern human forms, and discuss how these differences might illuminate aspects of modern human evolution.

## RESULTS

### The NAT1 gene in monkeys, apes, and humans

**Fig. 1.**
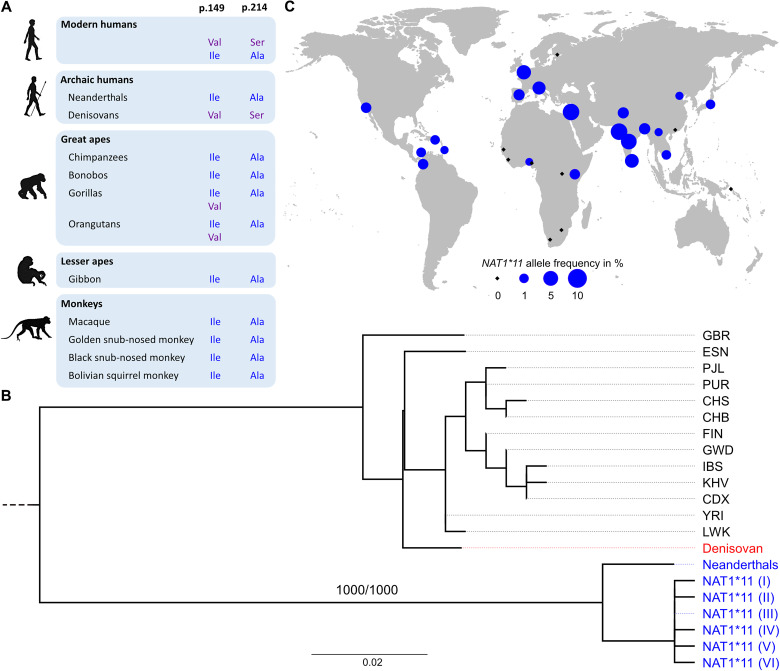
Evolutionary history of NAT1*11. (**A**) Amino acid residues at positions 149 and 214 of NAT1 across species. Created in BioRender. Fast, L. (2025) https://BioRender.com/t44ec03. (**B**) Phylogenetic tree of the 7526 bp-long sequence for individuals carrying the ancestral amino acids associated with the NAT1*11 haplotype, high-coverage archaic humans and noncarrier representatives from human populations in 1000 Genomes Project (1kGP). The tree is rooted using the inferred ancestral sequence. The Denisovan individual is shown in red, high-coverage Neanderthal genomes and present-day NAT1*11 carriers from 1kGP in blue, and all other modern humans in black. Numbers indicate branch support values, and the scale bar shows mutations per site. The tree is based on 75 segregating sites, including 16 singletons. Representatives of reference populations in 1kGP were randomly selected. Population abbreviations: PJL, Punjabi (Lahore, Pakistan); GBR, British from England and Scotland; IBS, Iberian populations (Spain); FIN, Finnish (Finland); PUR, Puerto Rican (Puerto Rico); KHV, Kin (Ho Chi Minh City, Vietnam); CDX, Chinese Dai (Xishuangbanna, China); CHB, Han Chinese (Beijing, China); CHS, Han Chinese South; LWK, Luhya (Webuye, Kenya); YRI, Yoruba (Ibadan, Nigeria); ESN, Esan (Nigeria); GWD, Gambian (Western Division, The Gambia – Mandinka). (**C**) Geographic distribution and allele frequency of rs4986783 in populations from phase 3 of 1kGP and in Bantu, San, Palestinian, and Papuan (Bougainville) populations from HGDP. Blue circles indicate the minor allele frequency in different populations worldwide, while black squares represent the absence of the ancestral allele.

To understand the evolutionary history of NAT1*11, we first examined reference genomes from several primates (Fig. 1A). All monkeys carry an isoleucine residue at position 149 (Ile^149^) and an alanine residue at position 214 (Ala^214^). The genome of the gibbon, a lesser ape, also encodes these amino acids, matching the NAT1*11 haplotype.

Next, we analyzed genomes from 79 great apes, including gorillas, orangutans, bonobos, and chimpanzees (*8*), together with the reconstructed ancestral states of the great ape clade. Across all nodes in the phylogeny, the two amino acids defining NAT1*11 are inferred to represent the ancestral state (table S2 and fig. S1). Accordingly, the two amino acid residues encoded by the human reference genome at positions 149 and 214 are derived. Notably, all great apes carry the ancestral alanine residue at position 214 (Ala^214^). At position 149, however, gorillas and orangutans carry either the ancestral isoleucine (Ile^149^) or the derived valine (Val^149^) (Fig. 1A and table S2). This variation among great apes has been previously reported (*9*). Although trans-species polymorphisms are rare, they are known to occur in primates (*10*, *11*). The divergence of the NAT1*11 haplotype, along with the clustering of Denisovans with modern humans (Fig. 1B), suggests deep evolutionary lineages at this locus.

We then analyzed genomes of the archaic humans Neanderthals and Denisovans. We found that the *NAT1* gene carried by all three high-coverage Neanderthals (*12*–*14*), and eight low-coverage Neanderthals (*12*, *15*–*20*), encodes the ancestral Ile^149^ and Ala^214^ (Fig. 1A and table S2). However, the Denisovan genome (*21*) encodes a NAT1 protein that is identical to the common modern human enzyme, including the derived valine and serine residues (Val^149^ and Ser^214^). The sharing of the derived state between modern humans and Denisovans suggests that these amino acids varied in the common ancestral population of archaic and modern humans.

### Neanderthal gene flow reintroduced the ancestral NAT1

The previous study reporting the two ancestral amino acids on a deeply divergent haplotype (NAT1*11) was based on 80 genomes, 4 of which carried the haplotype outside Africa (*6*). We reassessed the worldwide distribution of NAT1*11 using 2653 genomes from the 1000 Genomes Project [1kGP; (*22*)] and the Human Genome Diversity Project (*23*). We confirm the earlier finding that this haplotype is predominantly found outside Africa, reaching allele frequencies of up to 6.9% in South Asia, 4.4% in Europe, and 1.0% in East Asia, while being almost absent in sub-Saharan Africa (Fig. 1C and table S3).

Using the 1kGP dataset, we found that the two missense variants defining NAT1*11 always occur together [coefficient of determination (*R*^2^) = 1.0] and sit on a haplotype spanning 89 kb (*R*^2^ > 0.80). Some divergent haplotypes tens of kilobases long, mostly found outside Africa, exist in modern humans because of admixture with Neanderthals around 47,000 years ago (*24*–*26*). Such haplotypes should be more closely related to Neanderthal genomes than to other modern human haplotypes and too long to have persisted intact since humans and Neanderthals shared a common ancestor.

To determine whether NAT1*11 originates from Neanderthal introgression, we compared a 7.5-kb segment [containing the two missense variants in perfect linkage disequilibrium (LD), *R*^2^ = 1] of the 89-kb NAT1*11 haplotype with the genomes of 2590 modern humans, three Neanderthals, and one Denisovan. NAT1*11 proved most similar to Neanderthal genomes. Modern human sequences not carrying these ancestral amino acids formed a separate clade (Fig. 1B and fig. S2). Using multiple recombination maps, we estimate that the 89-kb NAT1*11 haplotype has a genetic length of 0.018 to 0.0731 cM (*27*) (table S4). In a recent dataset [1kGP; (*22*)], this length (using the shortest estimate of 0.018 cM) falls within the typical range for introgressed haplotypes from archaic humans, as it is longer than 41% of them (fig. S3). Given an assumed total branch length of 41,500 generations (*28*) and a published method (*29*), we find it unlikely that the NAT1*11 haplotype survived intact since the divergence of archaic and modern humans (*P* = 5.20 × 10^−3^ to 2.98 × 10^−12^; table S4). An analysis of sequence similarity between NAT1*11 and the archaic genomes shows that the introgressed fragment falls within the genetic variation of Neanderthals and is closest to the Vindija Neanderthals (fig. S4).

In summary, NAT1*11 has a geographic distribution typical of variants introduced by Neanderthals (Fig. 1C and table S3), closely resembles Neanderthal *NAT1* sequences (Fig. 1B and fig. S2), and is too long to have plausibly survived recombination since Neanderthals and modern humans diverged around 600,000 years ago (table S4) (*12*, *30*). We found evidence that the rare occurrences of this haplotype in individuals of African ancestry also derive from Neanderthal gene flow (table S5), most likely reflecting back-migration into Africa. We therefore conclude that NAT1*11 entered the modern human gene pool through gene flow from Neanderthals. At the same time, we do not exclude that other evolutionary forces, including balancing selection, may have shaped variation at this locus earlier in hominin evolution or in other primates.

Excluding gene flow, all modern humans carry two derived amino acid substitutions in the NAT enzyme, making it a rare example of a human protein in which more than one amino acid has been acquired and fixed. Such a pattern has been observed in only eight proteins genome-wide when excluding archaic reintroduction (*31*).

### The ancestral amino acid residues do not influence enzymatic activity of NAT1

We investigated the functional impact of the two amino acid substitutions at positions 149 and 214, both together and separately. We expressed the derived protein (Val-Ser) as a reference, along with the ancestral protein (Ile-Ala) and the single mutants (Val-Ala and Ile-Ser), in *Escherichia coli*. We then tested their activity using the substrate *p*-aminobenzoic acid (PABA), which loses fluorescence upon acetylation (*32*).

**Fig. 2.**
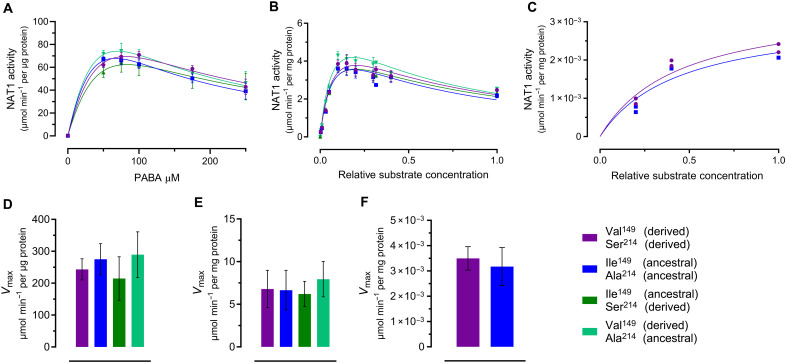
The ancestral amino acid residues do not affect NAT1 enzymatic activity. (**A**) Enzymatic activity of recombinant NAT1 variants expressed in *E. coli*, measured in the presence of 100 μM acetyl-CoA and varying concentrations of PABA (0 to 250 μM) using fluorometric detection. A substrate inhibition model was used to fit the dose-response curve, with *K*_m_ fixed at 101.9 μM. Independent biological replicates (*n* = 3) were performed, with error bars representing the SEM. (**B**) Enzymatic activity of recombinant NAT1 variants expressed in *E. coli*, measured in the presence of 100 μM acetyl-CoA and varying concentrations of a commercial substrate (Merck MAK430) (relative concentration: 0 to 1) using fluorometric detection. A substrate inhibition model was used to fit the dose-response curves. Independent biological replicates (*n* = 3) were performed, with error bars representing the SEM. (**C**) Enzymatic activity of recombinant NAT1 variants expressed in human Expi293 cells, measured in the presence of 100 μM acetyl-CoA and varying concentrations of a commercial substrate (Merck MAK430) (relative concentration: 0 to 1) using fluorometric detection. A Michaelis-Menten model was used to fit the dose-response curve, with *K*_m_ fixed at 0.4457. Because of low protein yield, only one biological replicate was performed. Two technical replicates per concentration are shown as symbols. (**D** to **F**) *V*_max_ values for NAT1 variants calculated from the dose-response curves in (A) to (C), respectively. Error bars represent the asymptotic 95% confidence intervals. Derived NAT1 (Val^149^, Ser^214^) is shown in purple, ancestral NAT1 (Ile^149^, Ala^214^) in dark blue, single mutant NAT1 (Ile^149^, Ala^214^) in green, and single mutant NAT1 (Val^149^, Ala^214^) in turquoise.

The resulting dose-response curves show a decrease in enzymatic activity at high substrate concentrations (Fig. 2A), suggesting a substrate inhibition model, as previously reported for this substrate (*33*). Because of technical issues with the fitting algorithm, we fixed the Michaelis-Menten constant (*K*_m_) as a shared value across datasets. The resulting *V*_max_ values were similar for all four enzyme variants (Fig. 2D). We also tested NAT1 activity using a substrate from a commercial kit (Merck MAK430) and again observed no differences in the dose-response curves (Fig. 2B) or *V*_max_ values among the four variants (Fig. 2E). Because bacterial expression systems may produce enzymes with different activity levels compared to mammalian cells because of posttranslational modifications (*34*, *35*), we also expressed the derived and ancestral proteins in human Expi293 cells. When tested with the kit substrate, the reaction velocities of the two variants produced in the mammalian cells similarly did not differ from each other (Fig. 2, C and F). Because of low protein yield, we were limited to two replicates.

### NAT1 enzymatic activity is reduced by the phosphomimetic Asp214

Using mass spectrometry, we investigated whether the NAT1 proteins expressed in human cells displayed any posttranslational modifications. We detected only N-terminal acetylation, which has previously been suggested to be transferred to NAT1 by the N-terminal acetyltransferase NatB (*36*). However, many modifications are cell type dependent and may be lost during protein preparation (*37*). While alanine, valine, and isoleucine residues generally do not undergo posttranslational modifications, serine residues can be phosphorylated.

**Fig. 3.**
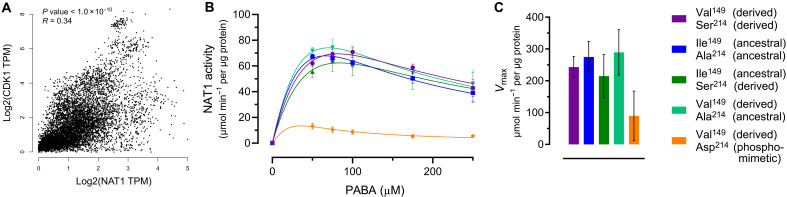
NAT1 enzymatic activity is reduced by phosphomimetic aspartate at position 214. (**A**) Correlation analysis of *NAT1* and *CDK1* expression across 8587 tissue samples from the GTEx database, performed using Gene Expression Profiling Interactive Analysis [GEPIA (*38*)]. TPM, transcripts per million. (**B**) Enzymatic activity of recombinant NAT1 variants expressed in *E. coli*, measured in the presence of 100 μM acetyl-CoA and varying concentrations of PABA (0 to 250 μM) using fluorometric detection. For comparison, the same data from Fig. 2A are shown, with the addition of NAT1 Val^149^ Asp^214^. A substrate inhibition model was used to fit the dose-response curve, with *K*_m_ fixed at 101.9 μM. Independent biological replicates (*n* = 3) were performed, with error bars representing the SEM. (**C**) *V*_max_ values for recombinant NAT1 variants expressed in *E. coli*, calculated from the dose-response curves in (B). Error bars represent the asymptotic 95% confidence intervals. Derived NAT1 (Val^149^, Ser^214^) is shown in purple, ancestral NAT1 (Ile^149^, Ala^214^) in dark blue, single-mutant NAT1 (Ile^149^, Ala^214^) in green, single-mutant NAT1 (Val^149^, Ala^214^) in turquoise, and phosphomimetic NAT1 (Val^149^, Asp^214^) in orange.

Predictions using NetPhos 3.1 suggest that cyclin-dependent kinase 1 (CDK1) (also known as cdc2) can phosphorylate Ser^214^ (fig. S5A). The prediction score of 0.57 falls within the range of scores for experimentally validated phosphorylation sites (fig. S5B). Expression of *CDK1* and *NAT1* correlates positively (*R* = 0.34, *P* < 1.0 × 10^−10^) across 8587 human tissue samples [Genotype-Tissue Expression Portal (GTEx); (*38*)], suggesting that NAT1 may be phosphorylated in vivo (Fig. 3A, correlation for individual tissues: table S6 and fig. S6). Previous studies have reported phosphorylation of Ser^214^ in breast cancer (*39*, *40*), lung adenocarcinoma (*41*), colon cancer, and normal adjacent tissue to colon cancers (*42*), supporting that Ser^214^ can be phosphorylated in vivo in tumor and in normal tissue.

To test whether phosphorylation of the derived Ser^214^ affects NAT1 activity, we replaced it with the negatively charged phosphomimetic amino acid aspartate (Asp^214^) (*43*, *44*) in the derived protein carrying Val^149^. We then expressed and purified the protein in *E. coli*. When tested in the PABA NAT1 activity assay alongside the four previously expressed *E. coli* proteins, the *V*_max_ of the phosphomimetic protein was reduced by 63% relative to the protein carrying Ser^214^ (Fig. 3, B and C).

### NAT1 residue 214 is located in the binding pocket entry

**Fig. 4.**
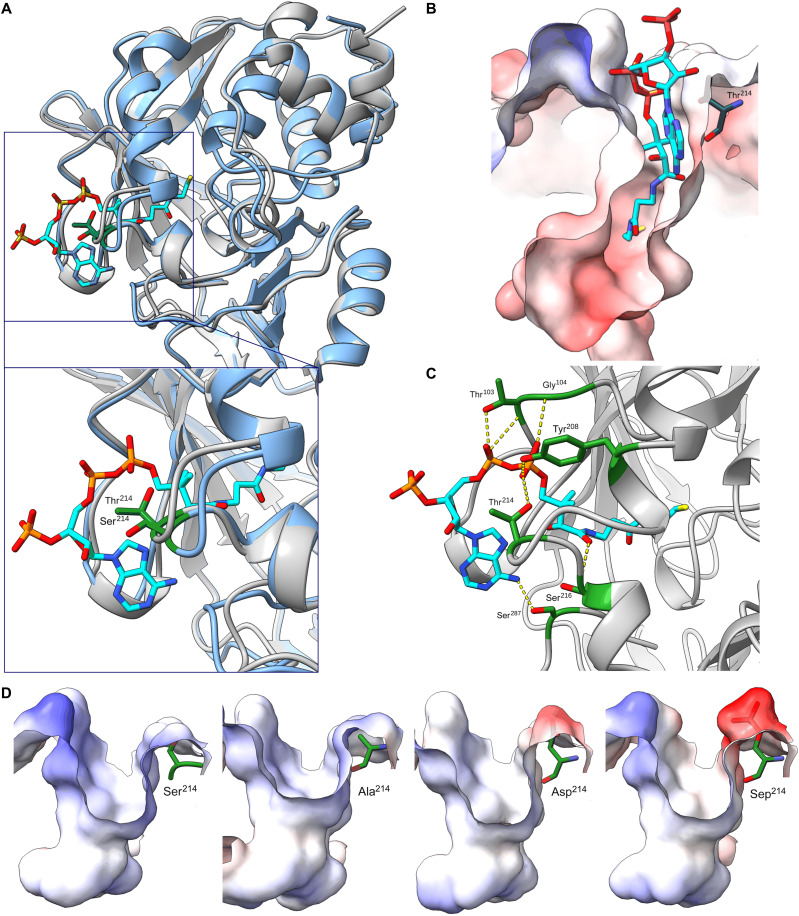
NAT1 residue 214 is located at the entry of the binding pocket. (**A**) Structural superimposition of the NAT1 crystal structure (light blue, PDB ID: 2PQT; (*45*)] and the NAT2 crystal structure in complex with CoA [gray, PDB ID: 2PFR; (*45*)]. Residue 214 is highlighted in green. The blue box provides a zoomed-in view of the binding pocket. Heteroatoms are labeled as follows: carbon (C), cyan; nitrogen (N), blue; phosphorus (P), orange; oxygen (O), red; sulfur (S), yellow. (**B**) Surface projection of the NAT2 binding pocket with bound CoA [PDB ID: 2PFR; (*45*)], colored by electrostatic potential (blue, positive; red, negative). Thr^214^ is highlighted in green. Heteroatoms are labeled as follows: C, cyan; N, blue; P, orange; O, red; S, yellow. (**C**) Crystallized structure of NAT2 in complex with bound CoA [PDB ID: 2PFR; (*45*)], showing a zoomed-in view of the binding pocket. Residues forming hydrogen bonds (yellow dashed lines) with CoA are highlighted in green. Heteroatoms are labeled as follows: C, cyan; N, blue; P, orange; O, red; S, yellow. (**D**) Surface projection of the NAT1 binding pocket, showing the electrostatic potential (blue, positive; red, negative) with the derived Ser^214^, ancestral Ala^214^, phosphomimetic Asp^214^, and phosphorylated Ser^214^ (Sep^214^).

We next investigated why phosphorylation of Ser^214^ might affect NAT1 activity. We used the published crystal structures of the derived NAT1 (Val^149^ and Ser^214^) bound to the inhibitor 2-bromoacetanilide [Protein Data Bank (PDB) ID: 2PQT] and its paralog NAT2 bound to CoA (PDB ID: 2PFR) (*45*). The structures of the two proteins are highly similar (root mean square deviation, 0.853 Å; sequence identity, 80.7%; Fig. 4A and fig. S7). In both proteins, residue 214 lies close to the CoA cofactor, on the surface near the entrance of the substrate-binding pocket (Fig. 4B). In NAT2, the corresponding residue (threonine) forms a hydrogen bond with the pyrophosphate group of CoA (Fig. 4C), suggesting that residue 214 in NAT1 may similarly influence CoA binding.

We used AlphaFold3 models (*46*) to predict binding-pocket residues in the ancestral and derived NAT1 proteins, as well as in proteins carrying the phosphomimetic Asp^214^ or phosphorylated Ser^214^. Residue 214 was consistently predicted to be located at the pocket entry (Fig. 4D). The negative charge introduced by phosphomimetic Asp^214^ or phosphorylated Ser^214^ could therefore affect substrate and CoA entry or exit at the catalytic site. Moreover, the surface location of Ser^214^ allows easy access for kinases or phosphatases (*47*), compatible with the idea that NAT1 activity may be regulated by phosphorylation of Ser^214^.

## DISCUSSION

NAT1 functions fall into two main categories: folate metabolism (*2*), which is important especially during development (*48*), and biotransformation of xenobiotics, including carcinogens (*49*, *50*). Using recombinantly expressed proteins, we demonstrate that modern humans are likely to have the ability to down-regulate NAT1 activity through phosphorylation of the serine residue at position 214. Because producing site-specifically phosphorylated proteins is technically challenging, we used a phosphomimetic aspartate. We acknowledge, however, that this substitution does not fully recapitulate all biochemical features of a phosphoserine (*51*). Previous studies assessing NAT1*11 activity have reported variable results (table S1). Differences in experimental design, including how posttranslational modifications such as phosphorylations are handled, may contribute to this variability.

One hypothesis for the importance of regulation of NAT1 activity could be its involvement in folate metabolism. During development, NAT1 is highly expressed in the neural tube (*52*), and NAT1-mediated acetylation of the folate catabolite p-aminobenzoylglutamate (pABGlu) is important for folate clearance (*2*, *53*). Therefore, higher NAT1 activity is associated with lower folate levels (*54*, *55*). Low folate levels increase the risk of birth defects (*56*), providing potential rationale for why *NAT1* haplotypes with lower activity have been associated with protection against developmental conditions such as spina bifida (*5*), limb anomalies (*57*), and cleft lip (*58*, *59*). These hypotheses remain to be tested in developmental models.

One measure of selection [Tajima’s D (*60*)] suggests that *NAT1* may have been under selection among modern humans (fig. S8). Since lower NAT1 activity may be beneficial under conditions of limited folate availability, this raises the question of whether folate intake decreased during modern human evolution. Two factors may have contributed to such a decrease. The first is a shift from a diet rich in fruits, leafy greens, and other plant-based sources of folate typical of nonhuman primates to a diet where folate-rich foods are less common (*61*). The second is the development of cooking, which reduces folate content in foods (*62*). Thus, it could be speculated that reduced folate intake might have made lower NAT1 activity beneficial, especially during pregnancy. Today, in industrialized regions, food is typically fortified with folate, and pregnant women take supplements to reduce the risk of birth defects (*56*), suggesting that natural dietary sources of folate may be insufficient.

Another role of NAT1 is the metabolism of xenobiotic aromatic and heterocyclic amines (*49*, *50*). While elevated NAT1 activity has been associated with increased risk to pancreatic cancer (*3*), higher NAT1 activity can also be beneficial. For example, higher NAT1 activity has been linked to a reduced risk of hypersensitivity reactions to the antibiotic sulfamethoxazole among individuals who are NAT2 slow acetylators (*63*), including carriers of the Neanderthal NAT1*11 haplotype.

The kinase CDK1 is predicted to phosphorylate Ser^214^ (fig. S5) and is coexpressed with *NAT1* in many tissues (Fig. 3A, table S6, and fig. S6). We note that *CDK1* is expressed primarily during development and that its expression declines sharply in many organs around birth (fig. S9). This suggests that NAT1 activity may be reduced by phosphorylation at Ser^214^ during embryonic development, potentially increasing folate levels. After birth, lack of phosphorylation might allow higher NAT1 activity, aiding detoxification of xenobiotics. We note that in addition to Denisovans that also carry Ser^214^, several other species have amino acid residues at this site that can either be phosphorylated or carry negative charges resembling phosphate groups (fig. S10). This might suggest adaptation of NAT1 activity not only in humans but also in other species.

We have not been able to associate traits with the Neanderthal NAT1*11 haplotype, although homozygous carriers of this variant would lack the ability to regulate NAT1 activity by phosphorylation at position 214. One reason for this might be the widespread folate fortification in Western diets. Future genetic studies, particularly in populations where the Neanderthal NAT1*11 haplotype is frequent, may uncover functional effects of the ancestral NAT1 enzyme.

## MATERIALS AND METHODS

### Length of the putative archaic segment and probability of haplotype block from shared ancestral lineage

For this study, LDProxy (*64*) was used to calculate the length of the haplotype found in carriers of the two missense SNPs that define NAT1*11. Data from all populations were analyzed using the latest high-coverage GRCh38 genome assembly.

Since the two missense variants are in perfect LD (*R*^2^ = 1), the length of the putative archaic segment in NAT1*11 carriers was determined by using the first variant (chromosome 8:18222492-G-A, GRCh38, rs4987076) as a proxy. The physical distance was then calculated between the last upstream and downstream variants with *R*^2^ > 0.8. This identified a segment of 89 kb, with rs28359515 (chr8:18218773, *R*^2^ = 0.9880) and rs148943016 (chr8:18307287, *R*^2^ = 0.8516) marking the haplotype boundaries.

To estimate the probability that an 89-kb haplotype originated from incomplete lineage sorting (ILS) in modern NAT1*11 carriers and Neanderthals, we applied the formula from Huerta-Sánchez *et al.* (*29*). This method first calculates the expected length of a shared ancestral sequence based on the local recombination rate and then determines the probability of observing a fragment of the given length at this locus.

Using a human lineage divergence time of 21,500 generations from Neanderthals and Denisovans, a Neanderthal lineage divergence of 19,500 generations (for a total branch length of 41,500 generations), and a genetic length of 0.018 cM [from the Icelandic sex-averaged recombination map in GRCh38; (*65*)], the probability of an 89-kb haplotype arising from ILS with Neanderthals is *P* = 5.2 × 10^−3^, supporting the hypothesis that this haplotype was introduced into the modern human gene pool via introgression.

However, since recombination has been breaking down haplotypes over the ~2000 generations since Neanderthal gene flow, haplotypes of varying lengths are expected in present-day human populations. A limitation of this method is that it assumes a single representative haplotype length, which does not fully capture the diversity of archaic segment lengths. We selected an *R*^2^ threshold of >0.8 to reflect the length of a putative archaic segment shared by most carriers of the coding variants in the NAT1*11 haplotype.

To calculate the length distribution of archaic segments, we used *hmmix*, a reference-free hidden Markov model that calls fragments by identifying genomic regions with an abundance of single-nucleotide variants not seen in an unadmixed outgroup population (*66*). We downloaded the called archaic segments in 1000 Genomes for all non-African populations from the GitHub page of the author in hg38 (https://github.com/LauritsSkov/Introgression-detection) and calculated the genetic length of each called fragment with a high-resolution Icelandic recombination map (*27*).

### Phylogenetic tree

To build the 7- and 89-kb haplotypes around *NAT1*, we merged a variant call format (VCF) with all sites called for the archaics, and all phased samples from the 1kGP VCF (*22*), and kept only segregating sites in callable regions of the loci we were interested in (www.internationalgenome.org/announcements/genome-accessibility-masks/). We further filtered out multiallelic sites and sites with missing data for any individual. To plot a single representative chromosome from the unphased archaics, we also removed sites that were heterozygous in archaics. We split the data into chromosomes and created fasta files for each individual in the merged VCF using VCF-KIT (https://vcf-kit.readthedocs.io/en/latest/, vk phylo fasta). For each tree, we kept the archaic haplotypes, the carriers of the NAT1*11 haplotype, and one representative noncarrier for each population in 1kGP. We then kept unique haplotypes and created maximum likelihood trees using IQ-TREE (https://academic.oup.com/mbe/article/37/5/1530/5721363) using bootstrapping of 1000 replicates.

### Data availability

The 1kGP is a publicly available whole-genome sequencing dataset (*22*). It provides a comprehensive overview of common human genetic diversity by sequencing a diverse set of individuals from various populations worldwide. The latest 1kGP release from 2022 (GRCh38) includes 2504 unrelated samples from 26 populations and 698 complete trios, totaling 3202 individuals, all sequenced at 30× coverage using Illumina technology. Data from 1kGP were downloaded from its FTP portal, and the 2022/04/22 release was used [(*67*); https://ftp.1000genomes.ebi.ac.uk/vol1/ftp/data_collections/1000G_2504_high_coverage/working]. From the 3202 samples, only those unrelated to any other participant were retained, on the basis of the text file provided in the repository (20130606_g1k_3202_samples_ped_population.txt). Samples with a listed mother or father ID were excluded, resulting in a final dataset of 2590 individuals. No further filtering was applied.

The Great Ape Genome Project generated high-coverage sequencing data for 79 wild- and captive-born individuals from all six great ape species and 10 subspecies across Africa and Southeast Asia (*8*). These data were downloaded from this repository (https://eichlerlab.gs.washington.edu/greatape/data/). Inferred ancestral states for the phylogenetic tree were obtained from the INFO fields of phase 3 of 1kGP (https://ftp.1000genomes.ebi.ac.uk/vol1/ftp/release/20130502/) and lifted over to GRCh38.

Sequences of the gibbon, macaque, and three monkeys (golden snub-nosed monkey, Bolivian squirrel monkey, and black snub-nosed monkey) for the two missense variants in NAT1 were retrieved from the 24 Primates EPO-Extended dataset via Ensembl Genome Browser 113 (rs4987076:

www.ensembl.org/Homo_sapiens/Variation/Compara_Alignments?db=core;r=8:18221992-18222992;v=rs4987076;vdb=variation;vf=543633621, rs4986783: www.ensembl.org/Homo_sapiens/Variation/Compara_Alignments?align=2050&db=core&r=8%3A18222187-18223187&v=rs4986783&vdb=variation&vf=543633606).

### Allele sharing analysis with archaic genomes

To assess allele sharing between modern and archaic humans across the 89-kb region encompassing *NAT1*, extended by 50 kb upstream and downstream, we generated a merged variant dataset including modern human genomes from the 1kGP and high-coverage archaic genomes. Before analysis, variants were intersected with regions of the genome that are strictly mappable, using the GRCh38 mappability mask provided by the 1kGP (https://ftp.1000genomes.ebi.ac.uk/vol1/ftp/data_collections/1000_genomes_project/working/20160622_genome_mask_GRCh38/), to minimize mapping and alignment artifacts. The archaic VCF contained all sites callable in the archaic genomes, including nonsegregating sites.

Following merging, we retained only biallelic single-nucleotide polymorphisms. Sites with missing genotypes in any individual were removed, and sites at which archaic genomes were heterozygous were excluded to enable representation of a single archaic haplotype in the allele-sharing plots. The resulting filtered dataset was used to quantify allele sharing and pairwise differences between modern human carriers and noncarriers of the NAT1*11 haplotype and archaic genomes across the LD-defined *NAT1* region.

### Selection analysis by Tajima’s D

We calculated Tajima’s D (*60*) to test departure from neutrality in the 200-kb region (chr8:18,150,000-18,350,000, GRCh38) surrounding *NAT1* and the putative Neanderthal haplotype. Tajima’s D provides unbiased results even in the presence of Neanderthal haplotypes in appreciable frequencies, which are much longer than older modern human haplotypes and would bias methods that depend on the length of the haplotype to infer selection. We used all phased haplotypes from the latest 30× coverage release of the 1kGP from 2022 (*67*) and kept biallelic single-nucleotide variants from the VCF. We used the R package PopGenome version 2.7.5 and moved in windows of 10 kb in steps of 1 and averaged the results for each step.

### Expression and purification of recombinant proteins

Recombinant NAT1 protein variants were produced at the Protein Science Facility, Karolinska Institutet, Stockholm. The NAT1 constructs NAT1*4 (derived, Val^149^, Ser^214^), NAT1*11 (ancestral, Ile^149^, Ala^214^), NAT1 Ile^149^ Ser^214^, NAT1 Val^149^ Ala^214^, and NAT1 Val^149^ Asp^214^, each carrying a His-tag, were transformed into *E. coli* BL21 (DE3) T1R pRARE2 cells. The cells were cultivated in Terrific Broth medium, and protein expression was induced with isopropyl-β-d-1-thiogalactopyranoside. The proteins were purified by immobilized metal affinity chromatography (IMAC), followed by size exclusion chromatography.

For NAT1*4 (derived, Val^149^, Ser^214^) and NAT1*11 (ancestral, Ile^149^, Ala^214^), the purified proteins were treated with tobacco etch virus (TEV) protease to remove the His-tag. The cleaved His-tag and His-tagged TEV protease were then removed by reverse IMAC purification. Because of inefficient cleavage, the His-tag was retained for the other constructs. Purified protein batches were aliquoted, flash frozen in liquid nitrogen, and stored at −80°C in 20 mM Hepes, 300 mM NaCl, 10% glycerol, and 2 mM Tris(2-carboxyethyl)phosphine (TCEP) (pH 7).

The NAT1*4 (derived, Val^149^, Ser^214^) and NAT1*11 (ancestral, Ile^149^, Ala^214^) constructs were also cloned into a pcDNA3.1 plasmid with an N-terminal TEV protease cleavage site, a Twin-Strep purification tag, a 3C protease cleavage site, and green fluorescent protein (GFP). Expi293 suspension cells were transfected using FectoPRO transfection reagent. In-cell expression levels were assessed by whole-cell GFP fluorescence imaging, and cells were harvested after 2 days. Proteins were purified on Strep-Tactin XT resin, and the GFP fusion was cleaved with TEV protease on-column. The cleaved proteins were further purified by reverse IMAC to remove the TEV protease. Purified batches were aliquoted, flash frozen in liquid nitrogen, and stored at −80°C in 100 mM tris-Cl, 150 mM NaCl, 1 mM EDTA, 50 mM biotin, 10 mM imidazole, 20% glycerol, and 2 mM TCEP (pH 8).

Total protein concentration was determined using the Bradford assay (Bio-Rad, 500-0006) (*68*). Protein purity and size were analyzed using the Bioanalyzer Protein 230 kit (Agilent Technologies, 5067-1517) according to the manufacturer’s instructions. For kinetic assays, the final NAT1 protein concentration was calculated on the basis of the proportion of correctly sized NAT1 protein relative to total protein, as purified samples contained traces of proteolytic degradation products or contaminating proteins.

### *N*-acetyltransferase activity assay

Recombinant NAT1 activity was tested in vitro using the Arylamine N-acetyltransferase Activity Assay Kit (Merck, MAK430). Each reaction contained either 0.25 μg (*E. coli*, PABA), 0.025 μg (*E. coli*, kit), or 1.6 μg (Expi293) of protein, along with 100 μM acetyl–coenzyme A (Merck, A2181), 2 mM dithiothreitol, and varying concentrations of 4-aminobenzoic acid (PABA) (0 to 250 μM, Merck, A9878) or NAT substrate I (relative concentration 0 to 1) provided in the kit. The reaction volume was 100 μl in NAT Assay Buffer, and reactions were performed in white flat-bottom 96-well plates (Thermo Fisher Scientific, 236105).

Samples were measured in duplicate at 37°C for 30 cycles using a CLARIOstar Plus plate reader (BMG Labtech) with the following fluorescence settings: NAT substrate I [excitation (Ex) = 360 nm/emission (Em) = 440 nm] and PABA (Ex = 270 nm/Em = 340 nm) (*32*). Enzymatic reaction rates were calculated as changes in relative fluorescence units (RFUs) over time. RFUs were converted to substrate concentration changes using standard curves for each substrate (fig. S11).

Dose-response curves were generated by plotting NAT1 activity against substrate concentration and fitting the data using either a Michaelis-Menten or substrate inhibition model in GraphPad Prism 10 (GraphPad Software Inc., La Jolla, CA), depending on the best fit. If the model was technically unstable, a global fit was first tested to determine whether one curve (i.e., a shared *K*_m_) could apply to all datasets. Because we could not reject the null hypothesis of no differences in K_m_ across conditions, we adopted this conservative assumption and fixed *K*_m_ to the shared estimate to enable stable estimation and comparison of *V*_max_ across datasets.

### Phosphorylation analysis of Ser^214^

Phosphorylation of NAT1 at Ser^214^ and of published experimentally verified phosphorylation sites was predicted using NetPhos 3.1 (*69*) with the full protein sequence from the UniProtKB (*70*). Expression correlation analysis between *NAT1* and *CDK1* was performed using Gene Expression Profiling Interactive Analysis (GEPIA), selecting all available GTEx tissues and calculating the Pearson correlation coefficient (*38*). Posttranslational modifications of NAT1 were searched in PhosphoSitePlus (*71*), dbPTM (*72*), PhosCancer (*73*), and PeptideAtlas (*74*).

### Structural analysis

*C*rystal structures of NAT1 [PDB ID: 2PQT; (*45*)] and NAT2 [PDB ID: 2PFR; (*45*)] were obtained from the PDB. Protein structures of NAT1 variants Ala^214^, Asp^214^, and Sep^214^ were predicted using AlphaFold3 (*46*) with high accuracy (pTM: 0.97, 0.97, and 0.96, respectively) using the same seed. The model with the highest pLDDT score was selected for further analysis.

Structural alignment was performed using UCSF ChimeraX 1.8 (*75*), which was also used to generate all protein structure images. Residues involved in pocket formation in NAT1 were predicted with CASTp 3.0 (*76*).

### Conservation analysis

Conservation of NAT1 protein (UniProt ID: P18440) was analyzed using ConSurf (*77*) with default parameters. The calculation was performed on a sample of 150 sequences that represent the list of homologues to the query. Conservation scores were calculated by Bayesian method, and amino acid substitution model was selected by best fit. For the multiple sequence alignment, protein sequences from UniProtKB (*70*) were used as input for alignment with Clustal Omega (*78*).

## Supplementary Materials

### This PDF file includes:

Figs. S1 to S11 Tables S1 to S6 References
