# Supplementary material for: Regulation of NAT1 activity in modern humans by a novel phosphorylation site
Source: Sci Adv. 2026 Jun 5;12(23):eady1666. doi: 10.1126/sciadv.ady1666 (PMC13240207; doi:10.1126/sciadv.ady1666)
Supplement: Supplementary file 1 — Figs. S1 to S11 Tables S1 to S6 References [file sciadv.ady1666_sm.pdf]

Science Advances
AAAS

Supplementary Materials for

Regulation of NAT1 activity in modern humans by a novel phosphorylation site

Luise Fast *et al.*

Corresponding author: Hugo Zeberg, hugo.zeberg@ki.se

Sci. Adv. **12**, eady1666 (2026)
DOI: 10.1126/sciadv.ady1666

This PDF file includes:

Figs. S1 to S11
Tables S1 to S6
References

	rs4987076	rs4986783
**Homo sapiens**	GGTGCCTTGTGTCTTCCGTTT	GACATCTCCATCATCTGTGTT
EPO: Pan-Homo	GGTGCCTTGTATCTTCCGTTT	GACATCTCCAGCATCTGTGTT
**Pan paniscus/troglodytes**	GGTGCCTTGTATCTTCCGTTT	GACATCTCCAGCATCTGTGTT
EPO: Gorilla-Homo	GGTGCCTTGTATCTTCCGTTT	GACATCTCCAGCATCTGTGTT
**Gorilla gorilla**	GGTTCCTTGTATCTTCTGTTT	GACATCTCCAGCATCTGTGTT
EPO: Pongo - Homo	GGTTCCTTGTATCTTCTGTTT	GACATCTCCAGCATCTGTGTT
**Pongo abelii**	GGTTCCTTGTATCTTCTGCTT	GACATCTCCAGCATCTGTGTT

Figure S1. Ancestral state reconstruction at rs4987076 and rs4986783. Alignment of the region surrounding rs4987076 and rs4986783 for all great apes, and the inferred EPO ancestral sequence for all lineages. Taken from Ensembl Release 115 (September 2025, https://www.ensembl.org/index.html). The missense variants are highlighted in blue (ancestral) and purple (derived).

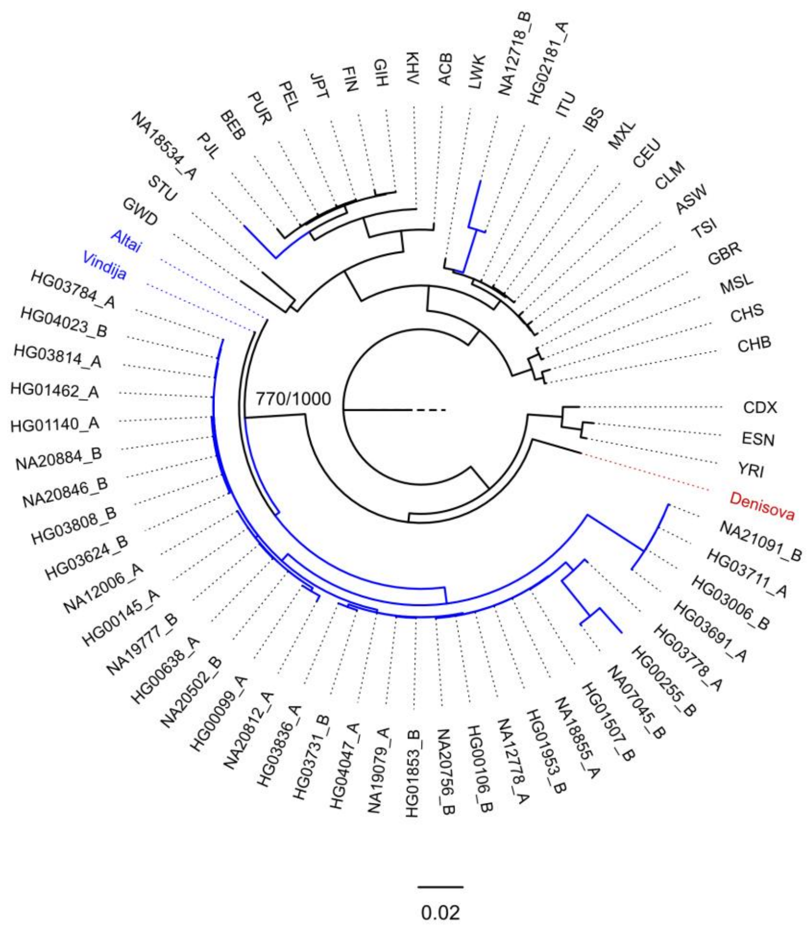

Figure S2. Phylogenetic tree for *NAT1*11*. Phylogenetic tree of the 89 kb-long sequence for individuals carrying the ancestral amino acids associated with the *NAT1*11* haplotype, high-coverage archaic humans and non-carrier representatives from human populations in 1kGP. The tree is rooted using the inferred ancestral sequence. High-coverage Neandertal genomes and their branches are shown in blue as well as chromosome branches of all modern humans carrying *NAT1*11*, the Denisovan individual is shown in red. All other modern humans are shown in black. Numbers indicate branch support values and the scale bar shows mutations per site. For the modern human carriers of *NAT1*11*, the chromosomes containing unique sequences are shown, with one representative selected at random. Three unique sequences cluster with modern humans due to recombination in a modern human background. Representatives of reference populations in 1kGP were randomly selected. Population abbreviations: GIH, Gujarati Indians (Houston, Texas, USA); ITU, Indian Telugu (UK); STU, Sri Lankan Tamil (UK); PJL, Punjabi (Lahore, Pakistan); BEB, Bengali (Bangladesh); GBR, British from England and Scotland; TSI, Toscani (Italy); CEU, Utah residents (CEPH) with Northern and Western European ancestry; IBS, Iberian populations (Spain); FIN, Finnish (Finland); MXL, Mexican Ancestry (Los Angeles, California, USA); CLM, Colombian (Medellin, Colombia); PEL, Peruvian (Lima, Peru); PUR, Puerto Rican (Puerto Rico); KHV, Kin (Ho Chi Minh City, Vietnam); JPT, Japanese (Tokyo), CDX, Chinese Dai (Xishuangbanna, China); CHB, Han Chinese (Beijing, China); CHS, Han Chinese South; LWK, Luhya (Webuye, Kenya); ACB, African Caribbean (Barbados); YRI, Yoruba (Ibadan, Nigeria); ASW, African Ancestry (Southwest US); ESN, Esan (Nigeria); GWD, Gambian (Western Division, The Gambia – Mandinka); MSL, Mende (Sierra Leone).

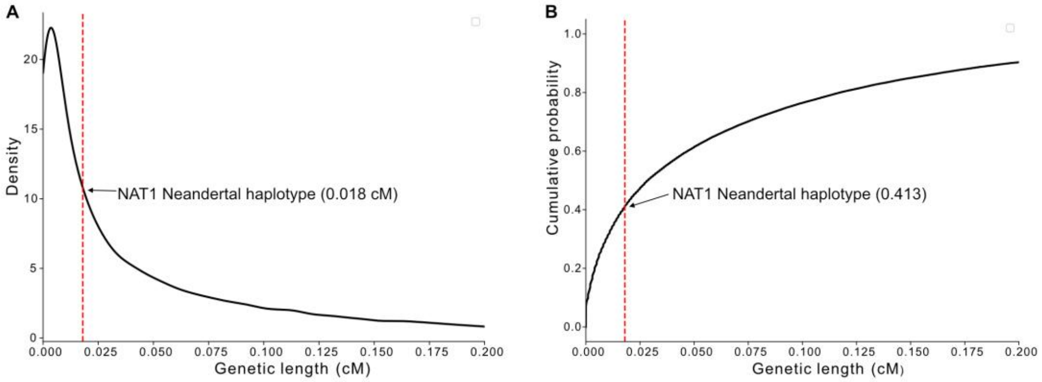

Figure S3. Probability density and cumulative distribution function of introgressed Neandertal haplotypes. **(**A) Probability density function of all archaic segments identified in out-of-Africa populations in 1kGP using *hmmix*. (B) Cumulative distribution function of A. The genetic length of the *NAT1* haplotype is highlighted with a red dotted line.

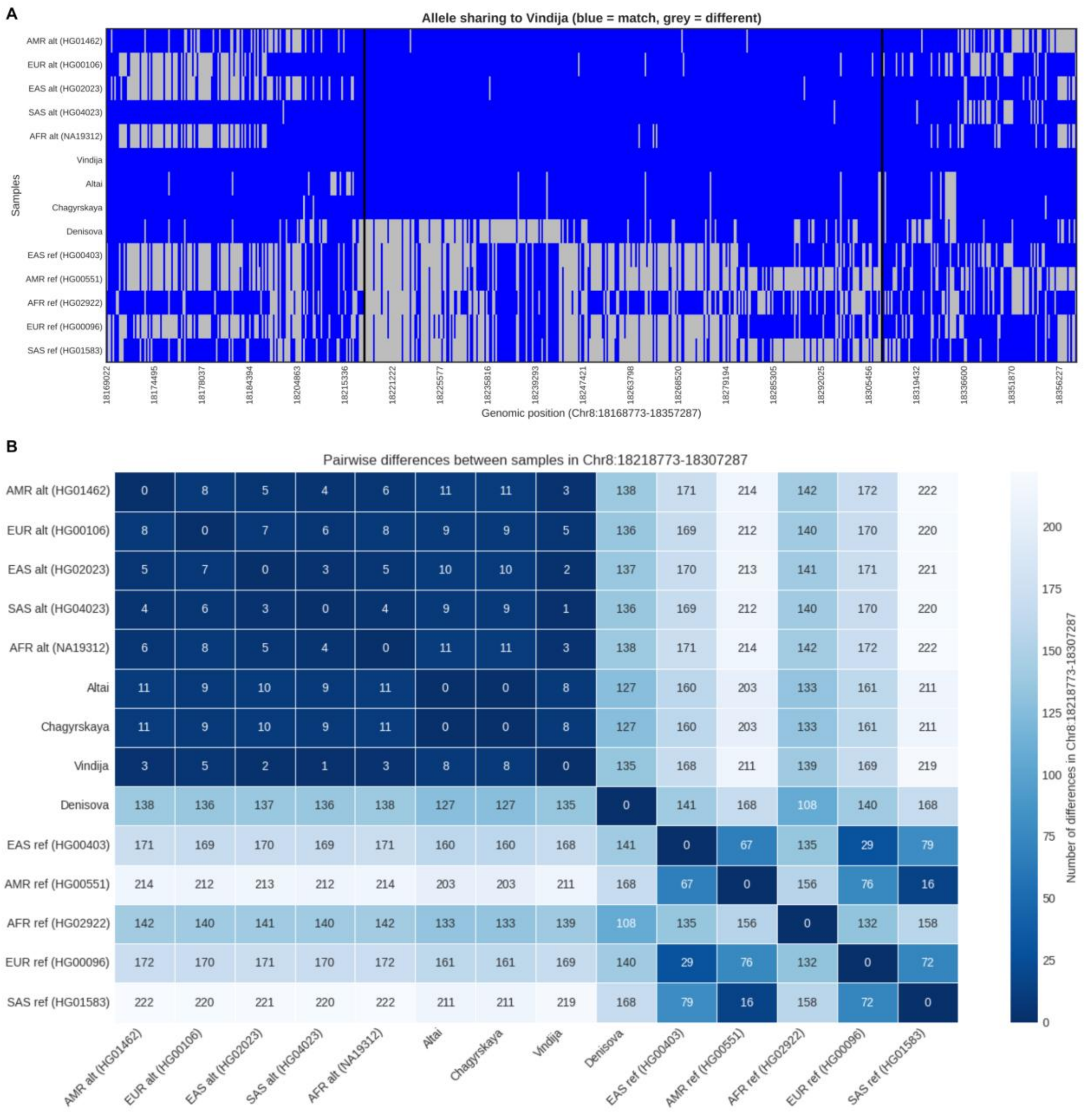

Figure S4. Allele sharing between the Vindija 33.19 Neandertal and modern human carriers as well as non-carriers of the *NAT1*11* haplotype. (A) Sharing of alleles with Vindija for modern human carriers of the *NAT1*11* haplotype (first 5 rows), other archaic humans (Vindija, Altai, Chagyrskaya, Denisova) and non-carriers of the *NAT1*11* haplotype (last 5 rows). We chose one carrier and non-carrier per superpopulation, and selected biallelic sites with no missingness in the 89 kb region defined by a linkage of $R^2 > 0.8$ to rs4986783, plus 50 kb upstream and downstream. We removed sites where the archaic genomes are heterozygous to plot a single representative chromosome for each of them. Alleles shared with Vindija are depicted in dark blue and alleles that are different in grey. (B) Heatmap matrix with pairwise differences between modern human carriers, non-carriers and archaic humans in the 89 kb region.

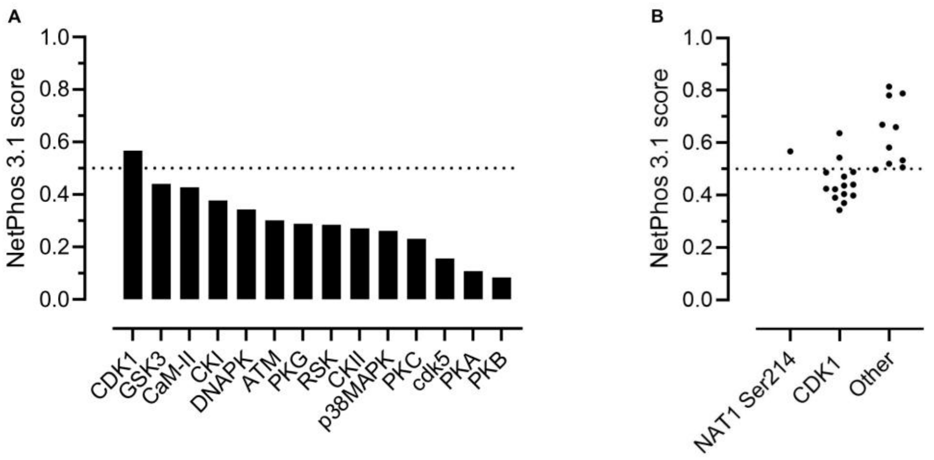

Figure S5. Phosphorylation prediction scores by NetPhos 3.1 (*69*). (A) NetPhos 3.1 prediction of the phosphorylation of NAT1 Ser214 by different kinases. A NetPhos 3.1 score above 0.5 (dashed line) is indicative of a positive prediction. (B) NetPhos 3.1 prediction scores for phosphorylation sites described in the literature. NAT1 Ser214 is predicted to be phosphorylated by CDK1, with a score of 0.57. 'CDK1' refers to predictions for 14 known phosphorylation sites that are phosphorylated by CDK1. 'Other' refers to the 10 phosphorylation sites described in the literature where NetPhos predicts the same kinase as the one reported in the literature.

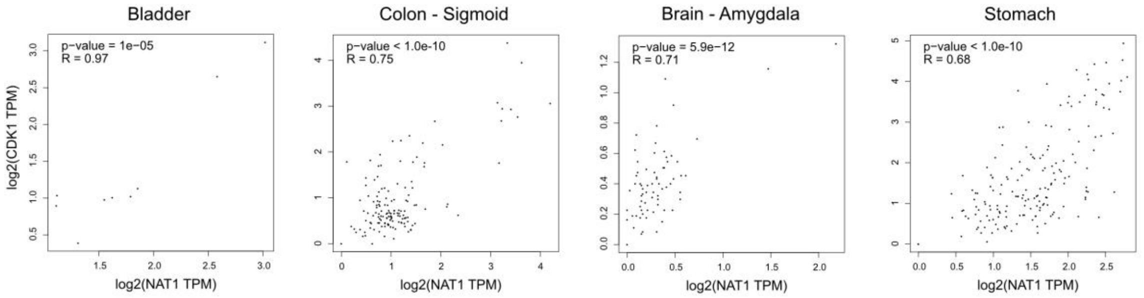

Figure S6. Correlation analysis of *NAT1* and *CDK1* expression. *NAT1* and *CDK1* co-expression was analysed using GEPIA (*38*). GTEx tissues with the highest Pearson's correlation coefficient (R) among the 54 tissues analysed (Table S6) are shown. TPM = transcripts per million.

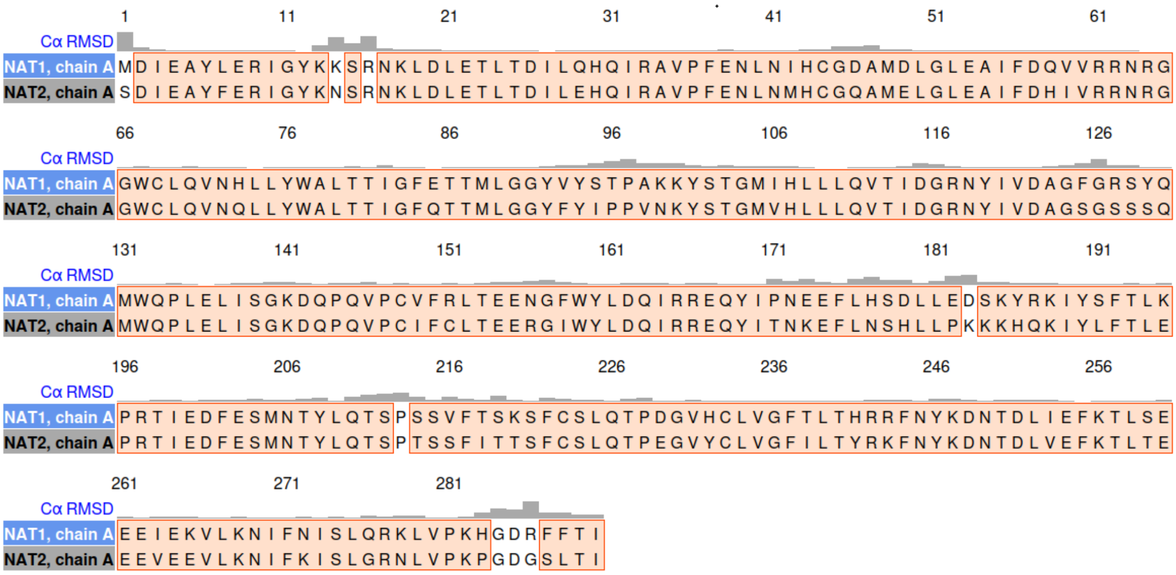

Figure S7. Structural sequence alignment of NAT1 and NAT2. The shown sequence alignment is based on the structural superimposition of NAT1 (blue) and NAT2 (grey) in figure 4A. Structurally conserved regions are highlighted in orange and the root-mean-square deviation (RMSD) for the alpha-carbons is indicated above the sequence. Bar height corresponds to RMSD values ranging from 0.05 Å to 6.69 Å.

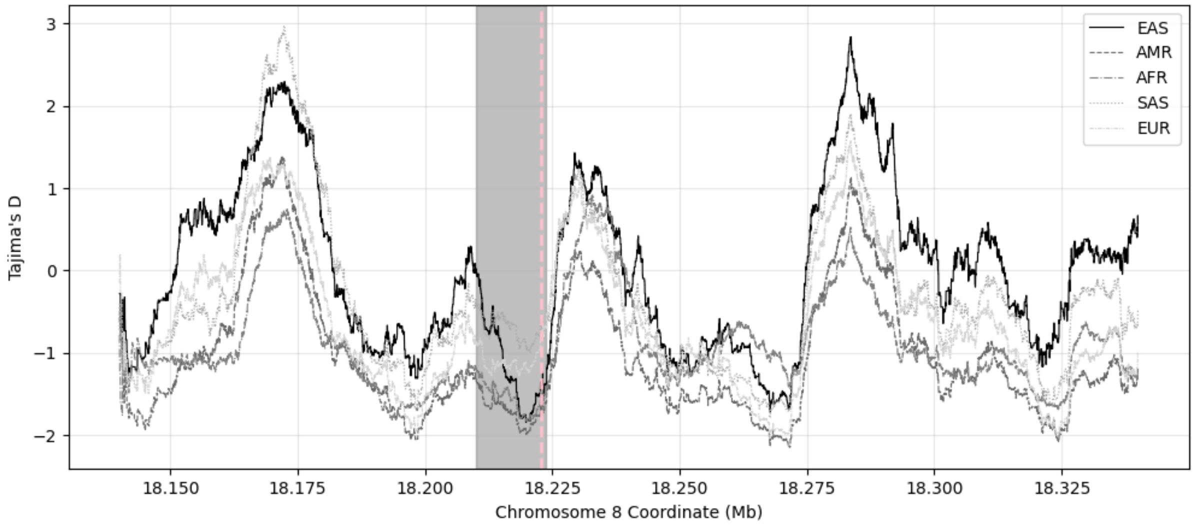

Figure S8. Tajima's D in different superpopulations in 1kGP in the region of the *NAT1* gene (hg38, chr8:18,150,000-18,350,000). The pink dotted line indicates the position of rs4986783, and the shaded area corresponds to the transcript of *NAT1* including UTRs (hg38 chr8:18,210,109-18,223,689). EAS (East Asian), AMR (Admixed American), AFR (African), SAS (South Asian), and EUR (European).

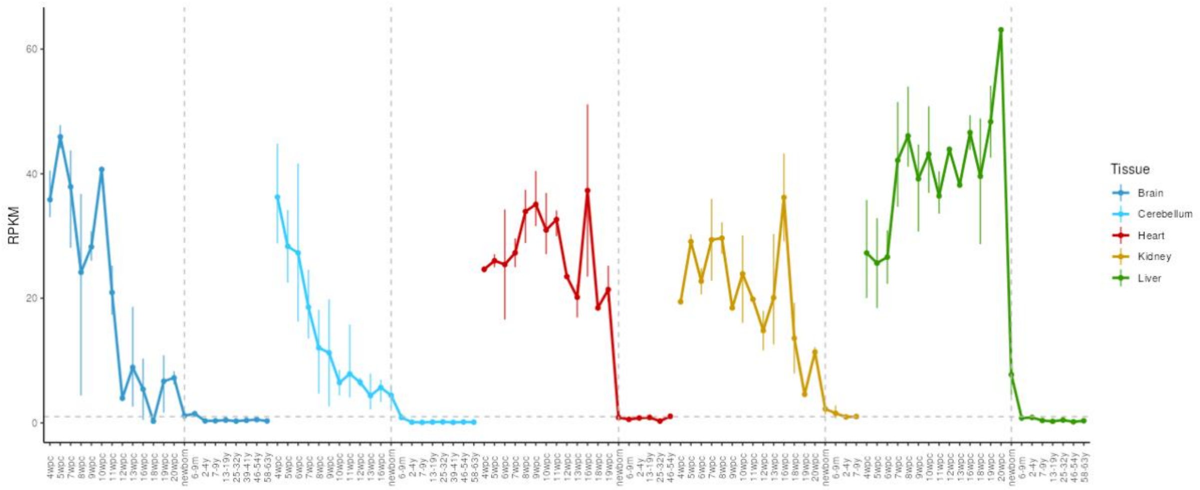

Figure S9. Gene expression of kinase *CDK1* during human development. Expression in reads per kilobase of transcript per million mapped reads (RPKM) is shown for different organs (see legend) across developmental stages. The vertical dashed lines indicate the newborn. Data are taken from the Evo-devo mammalian organs database and can be accessed under https://apps.kaessmannlab.org/evodevoapp/ (*91*).

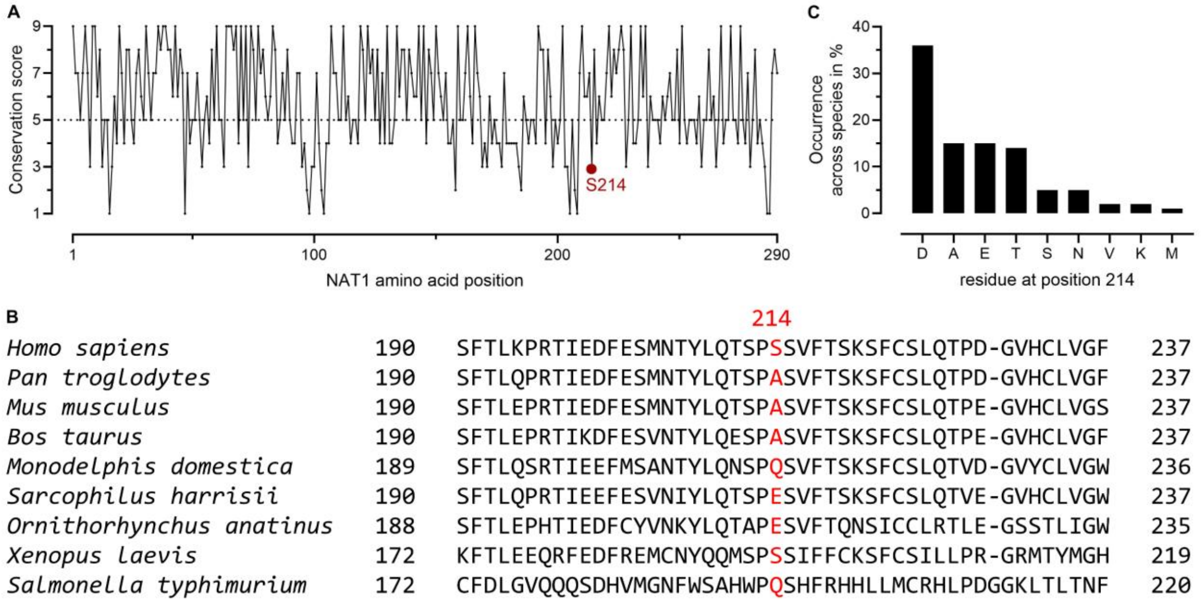

Figure S10. Conservation analysis of NAT1 residue 214. (A) Conservation score of NAT1 calculated by ConSurf (*77*) for 150 NAT1 homologues. The score ranges from 1 (low conservation) to 9 (high conservation). (B) Multiple sequence alignment of NAT1 around residue 214 (highlighted in red) across species. The numbers indicate the position of the amino acids in the protein. Placental mammals (*Eutheria*): *Homo sapiens* (Human), *Pan troglodytes* (Chimpanzee), *Mus musculus* (House mouse), *Bos taurus* (Cow). Marsupial (*Metatheria*): *Monodelphis domestica* (Gray short-tailed opossum), *Sarcophilus harrisii* (Tasmanian devil). Monotreme (*Protheria)*: *Ornithorhynchus anatinus* (Platypus). Amphibia (non-mammalian vertebrate): *Xenopus laevis* (African clawed frog). Prokaryote: *Salmonella enterica* (bacterium). (C) 150 homologous sequences of NAT1 were analysed using ConSurf (*69*) and the amino acid residue aligned to position 214 of human NAT1 was extracted. D - aspartate, A - alanine, E - glutamate, T - threonine, S - serine, N - asparagine, V - valine, K - lysine, M - methionine.

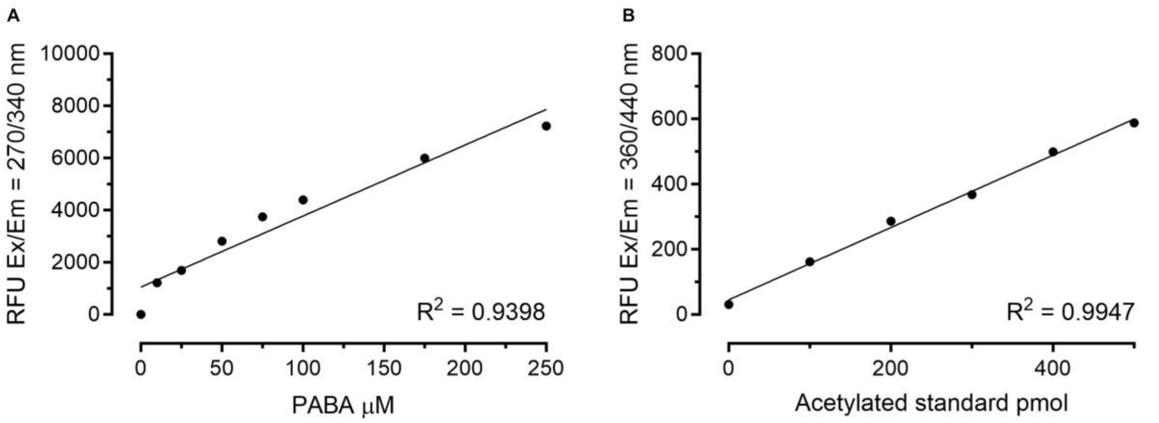

Figure S11. Standard curves for NAT1 Activity Assays. (A) Standard curve for PABA. Relative fluorescent units (RFU) for NAT1 Activity Assay reaction mix were measured without enzyme present for varying amounts of PABA substrate (0-250 μM) by fluorometric measurements at Ex/Em = 270/340 nm. A linear regression was fitted using GraphPad Prism. Every dot represents the mean of 3 technical replicates and the error bars show the s.e.m. (B) Standard curve for acetylated standard of NAT activity assay kit MAK430. Relative fluorescent units (RFU) for NAT1 Activity Assay reaction mix were measured without enzyme present for varying amounts of acetylated standard (0-500 pmol) by fluorometric measurements at Ex/Em = 360/440 nm. A linear regression was fitted using GraphPad Prism. Every dot represents the mean of 2 technical replicates and the error bars show the s.e.m.

Table S1. NAT1*11 enzymatic activity for N-acetylation compared to most common reference (NAT1*4) across studies. PAS – *p*-aminosalicylic acid, PABA – *p*-aminobenzoic acid, INH - isonicotinic acid hydrazide.

	Study	Protein	Substrate(s)	Sample size/Further comments
**Equal activity**	Hughes et al. 1998 (*79*)	recombinant (*E. coli*) isolated (human urine)	PAS	6 heterozygous NAT1*11 carriers tested
	De Leon et al. 2000 (*80*)	recombinant (*E. coli*, COS-1)	PAS, PABA, 5-aminosalicylate, 2-aminofluorene	
	Vaziri et al. 2000 (*81*)	isolated (human bladder and whole blood lysates)	PAS	5 heterozygous NAT1*11 carriers tested
	Fretland et al. 2001 (*82*)	recombinant (yeast)	PABA	
	Fretland et al. 2002 (*83*)	recombinant (yeast)	4-aminobiphenyl	
	Wang et al. 2011 (*63*)	isolated (HEK293, human B-lymphocytes)	PABA	increased NAT1*11 activity in lymphocytes from heterozygous carriers, but presumably due to increased protein expression
**Increased activity**	Doll et al. 1997 (*84*)	recombinant (*E. coli*)	PABA, PAS, and INH	only NAT1*4 Val149Ile tested
	Zhu and Hein 2008 (*85*)	recombinant (COS-1)	PABA	
**Lower activity**	Risch et al. 1996 (*86*)	isolated (human erythrocytes)	PABA	1 heterozygous NAT1*11 carrier tested
	Payton et al. 1998 (*87*)	isolated (human erythrocytes)	PABA	1 heterozygous NAT1*11 carrier tested
	Bruhn et al. 1999 (*88*)	isolated (human red blood cells)	PABA	8 heterozygous and 2 homozygous NAT1*11 carriers tested
	Zhangwei et al. 2006 (*89*)	isolated (human leukocytes)	PABA	1 homozygous, 1 heterozygous NAT1*11 carrier tested

Table S2. Allele frequencies at rs4987076 and rs4986783 in great apes and modern *Homo sapiens*, and the genotypes of ancient modern humans and the high- and low-coverage Neandertals and Denisovans. Allele frequencies are taken from the VCF made available in Prado-Martinez et al. 2013 (*8*). Individuals with the same genotype are grouped together.

Allele frequencies

		rs4987076		rs4986783	
		**Ref (G)**	**Alt (A)**	**Ref (T)**	**Alt (G)**
**Great apes**	Pan paniscus	0.26	0.74	0	1
	Pan troglodytes	1	1	0	1
	Pongo abelii	1	1	0	1
	Pongo pygmaeus	0.6	0.4	0	1
	Gorilla gorilla	0.4	0.6	0	1
**Homo sapiens**		0.98	0.02	0.98	0.02

Genotypes

		rs4987076	rs4986783
**Archaic humans**	High-coverage Neandertal genomes (Altai, Vindija, Chagyskaya)	1/1 (A/A)	1/1 (G/G)
	Low-coverage Neandertal genomes (Mezmaiskaya1, Mezmaiskaya2, VindijaG1, Spy, Scladina, Hohlenstein-Stadel, Forbes Quarry, Les Cottes)	1/1 (A/A)	1/1 (G/G)
	Denisovan genomes (Denisova 3, Denisova 25)	0/0 (G/G)	0/0 (T/T)
**Ancient modern humans**	Ust’-Ishim	0/1 (G/A)	0/1 (T/G)
	Sunghir3, Loshbour, Yana1, Yana2, Kolymal	0/0 (G/G)	0/0 (T/T)

Table S3. Frequency of *NAT1*11* for all Superpopulations and Populations in 1kGP.

Superpopulation	Population	Haplotype Count	Allele Number	Number of homozygotes	Allele Frequency
South Asian	**Overall**	**42**	**1028**	**3**	**0.041**
	Gujarati Indians in Houston, Texas, USA (GIH)	14	204		0.069
	Indian Telugu in the UK (ITU)	12	208	3	0.058
	Sri Lankan Tamil in the UK (STU)	8	206		0.039
	Punjabi in Lahore, Pakistan (PJL)	4	208		0.019
	Bengali in Bangladesh (BEB)	4	202		0.02
European	**Overall**	**27**	**1050**	**0**	**0.026**
	British from England and Scotland (GBR)	8	180		0.044
	Toscani in Italy (TSI)	7	214		0.033
	Utah residents (CEPH, USA) with Northern and Western European ancestry (CEU)	8	244		0.033
	Iberian populations in Spain (IBS)	4	214		0.019
	Finnish in Finland (FIN)	0	198		0
American	**Overall**	**9**	**706**	**0**	**0.013**
	Mexican Ancestry in Los Angeles, California, USA (MXL)	2	130		0.015
	Colombian in Medellin, Colombia (CLM)	3	194		0.015
	Peruvian in Lima, Peru (PEL)	2	174		0.011
	Puerto Rican in Puerto Rico (PUR)	2	208		0.01
East Asian	**Overall**	**6**	**1024**	**0**	**0.006**
	Kin in Ho Chi Minh City, Vietnam (KHV)	2	202		0.01
	Japanese in Tokyo, Japan (JPT)	2	208		0.01
	Chinese Dai in Xishuangbanna, China (CDX)	1	184		0.005
	Han Chinese in Beijing, China (CHB)	1	206		0.005
	Han Chinese South, China (CHS)	0	224		0
African	**Overall**	**5**	**1372**	**0**	**0.004**
	Luhya in Webuye, Kenya (LWK)	3	194		0.015
	African Caribbean in Barbados (ACB)	1	192		0.005
	Yoruba in Ibadan, Nigeria (YRI)	1	240		0.004
	African Ancestry in Southwest US (ASW)	0	122		0
	Esan in Nigeria (ESN)	0	212		0
	Gambian in Western Division, The Gambia - Mandinka (GWD)	0	238		0
	Mende in Sierra Leone (MSL)	0	174		0
**Overall**		**89**	**5180**	**3**	**0.017**

Table S4. Genetic length (cM) and probability of incomplete lineage sorting of the 89kb *NAT1* haplotype (chr8:18218773-18307287) using different genetic maps from https://www.chg.ox.ac.uk/~anjali/AAmap/.

	deCODE (hg38)	combinedLD	AA map	YRI	CEU	African enriched
**Genetic length (cM)**	0.018	0.0522	0.0486	0.0731	0.0241	0.0248
**Probability P**	5.2×10^{-3}	1.14×10^{-8}	4.64×10^{-8}	2.98×10^{-12}	5.56×10^{-4}	4.29×10^{-4}

Table S5. Neanderthal segments identified by IBDmix (*90*) on African carriers of *NAT1*11*. The start and length of the Neandertal segments in Africans are very similar to the introgressed segments found in non-Africans.

Chr	Start	End	Size (kb)	Population	Ancestry	ID
8	18074464	18164238	89.77	ACB	AFR	HG02009
8	18074464	18164238	89.77	LWK	AFR	NA19312
8	18075965	18164238	88.27	LWK	AFR	NA19475
8	18075999	18164238	88.23	LWK	AFR	NA19448

Table S6. Correlation analysis of *NAT1* and *CDK1* expression across 54 tissues from the GTEx database, performed using GEPIA (*38*). Tissues are ranked by the Pearson's correlation coefficient (R).

Tissue	R	p-value
Bladder	0.97	1.00E-05
Colon – Sigmoid	0.75	<1.00E-10
Brain – Amygdala	0.71	5.90E-12
Stomach	0.68	<1.00E-10
Cells – Leukemia cell line (CML)	0.66	5.70E-10
Esophagus – Gastroesophageal Junction	0.64	<1.00E-10
Muscle – Skeletal	0.56	<1.00E-10
Cervix – Endocervix	0.55	0.45
Colon – Transverse	0.53	1.10E-13
Heart – Left Ventricle	0.52	1.60E-15
Esophagus – Muscularis	0.51	<1.00E-10
Brain – Nucleus accumbens (basal ganglia)	0.49	1.40E-07
Brain – Hypothalamus	0.48	5.20E-06
Brain – Anterior cingulate cortex	0.46	1.50E-05
Esophagus – Mucosa	0.45	4.00E-15
Minor Salivary Gland	0.45	5.10E-04
Brain – Substantia nigra	0.42	1.20E-03
Small Intestine – Terminal Ileum	0.41	6.10E-05
Brain – Caudate (basal ganglia)	0.37	6.60E-05
Pancreas	0.37	1.10E-06
Artery – Aorta	0.36	9.30E-08
Ovary	0.35	7.80E-04
Artery – Coronary	0.34	1.40E-04
Vagina	0.34	1.40E-03
Artery – Tibial	0.33	1.40E-08
Cells – EBV-transformed lymphocytes	0.32	7.60E-04
Kidney – Cortex	0.27	0.16
Nerve – Tibial	0.27	3.60E-06
Brain – Hippocampus	0.26	0.02
Adrenal Gland	0.25	4.70E-03
Brain – Frontal Cortex (BA9)	0.23	0.02
Lung	0.23	5.80E-05
Testis	0.23	3.00E-03
Skin – Sun Exposed (Lower leg)	0.22	5.60E-05
Thyroid	0.22	2.10E-04
Brain – Cerebellar Hemisphere	0.21	0.04
Brain – Spinal cord (cervical c-1)	0.18	0.17
Heart – Atrial Appendage	0.18	0.02
Spleen	0.17	0.09
Brain – Cortex	0.16	0.11
Brain – Cerebellum	0.14	0.13
Whole Blood	0.11	0.05
Brain – Putamen (basal ganglia)	0.11	0.35

Fallopian Tube	0.11	0.86
Liver	0.10	0.32
Skin – Not Sun Exposed (Suprapubic)	0.07	0.29
Adipose – Visceral (Omentum)	0.07	0.34
Adipose – Subcutaneous	0.06	0.28
Prostate	-0.01	0.92
Pituitary	-0.09	0.38
Breast – Mammary Tissue	-0.09	0.21
Uterus	-0.15	0.20
Cells – Transformed fibroblasts	-0.30	8.30E-07
Cervix – Ectocervix	-0.48	0.34

REFERENCES

1. D. W. Hein, Acetylator genotype and arylamine-induced carcinogenesis. *Biochim. Biophys. Acta* **948**, 37–66 (1988).

2. R. F. Minchin, Acetylation of p-aminobenzoylglutamate, a folic acid catabolite, by recombinant human arylamine N-acetyltransferase and U937 cells. *Biochem. J.* **307**, 1–3 (1995).

3. K. Zhang, L. Gao, Y. Wu, J. Chen, C. Lin, S. Liang, J. Su, J. Ye, X. He, NAT1 polymorphisms and cancer risk: a systematic review and meta-analysis. *Int. J. Clin. Exp. Med.* **8**, 9177–9191 (2015).

4. L. E. Jensen, K. Hoess, A. S. Whitehead, L. E. Mitchell, The NAT1 C1095A polymorphism, maternal multivitamin use and smoking, and the risk of spina bifida. *Birth Defects Res. A Clin. Mol. Teratol.* **73**, 512–516 (2005).

5. L. E. Jensen, K. Hoess, L. E. Mitchell, A. S. Whitehead, Loss of function polymorphisms in NAT1 protect against spina bifida. *Hum. Genet.* **120**, 52–57 (2006).

6. E. Patin, L. B. Barreiro, P. C. Sabeti, F. Austerlitz, F. Luca, A. Sajantila, D. M. Behar, O. Semino, A. Sakuntabhai, N. Guiso, B. Gicquel, K. McElreavey, R. M. Harding, E. Heyer, L. Quintana-Murci, Deciphering the ancient and complex evolutionary history of human arylamine N-acetyltransferase genes. *Am. J. Hum. Genet.* **78**, 423–436 (2006).

7. S. Boukouvala, The Database of Arylamine N-Acetyltransferases (NATs) (2025); https://nat.mbg.duth.gr/Human_NAT1_alleles.htm.

8. J. Prado-Martinez, P. H. Sudmant, J. M. Kidd, H. Li, J. L. Kelley, B. Lorente-Galdos, K. R. Veeramah, A. E. Woerner, T. D. O'Connor, G. Santpere, A. Cagan, C. Theunert, F. Casals, H. Laayouni, K. Munch, A. Hobolth, A. E. Halager, M. Malig, J. Hernandez-Rodriguez, I. Hernando-Herraez, K. Prufer, M. Pybus, L. Johnstone, M. Lachmann, C. Alkan, D. Twigg, N. Petit, C. Baker, F. Hormozdiari, M. Fernandez-Callejo, M. Dabad, M. L. Wilson, L. Stevison, C. Camprubi, T. Carvalho, A. Ruiz-Herrera, L. Vives, M. Mele, T. Abello, I. Kondova, R. E. Bontrop, A. Pusey, F. Lankester, J. A. Kiyang, R. A. Bergl, E. Lonsdorf, S. Myers, M. Ventura,

P. Gagneux, D. Comas, H. Siegismund, J. Blanc, L. Agueda-Calpena, M. Gut, L. Fulton, S. A. Tishkoff, J. C. Mullikin, R. K. Wilson, I. G. Gut, M. K. Gonder, O. A. Ryder, B. H. Hahn, A. Navarro, J. M. Akey, J. Bertranpetit, D. Reich, T. Mailund, M. H. Schierup, C. Hvilsom, A. M. Andres, J. D. Wall, C. D. Bustamante, M. F. Hammer, E. E. Eichler, T. Marques-Bonet, Great ape genetic diversity and population history. *Nature* **499**, 471–475 (2013).

9. C. Vangenot, P. Gagneux, N. G. de Groot, A. Baumeyer, M. Mouterde, B. Crouau-Roy, P. Darlu, A. Sanchez-Mazas, A. Sabbagh, E. S. Poloni, Humans and chimpanzees display opposite patterns of diversity in arylamine N-acetyltransferase genes. *G3* **9**, 2199–2224 (2019).

10. L. Segurel, E. E. Thompson, T. Flutre, J. Lovstad, A. Venkat, S. W. Margulis, J. Moyse, S. Ross, K. Gamble, G. Sella, C. Ober, M. Przeworski, The ABO blood group is a trans-species polymorphism in primates. *Proc. Natl. Acad. Sci. U.S.A.* **109**, 18493–18498 (2012).

11. J. C. Teixeira, C. de Filippo, A. Weihmann, J. R. Meneu, F. Racimo, M. Dannemann, B. Nickel, A. Fischer, M. Halbwax, C. Andre, R. Atencia, M. Meyer, G. Parra, S. Paabo, A. M. Andres, Long-term balancing selection in LAD1 maintains a missense trans-species polymorphism in humans, chimpanzees, and bonobos. *Mol. Biol. Evol.* **32**, 1186–1196 (2015).

12. K. Prufer, C. de Filippo, S. Grote, F. Mafessoni, P. Korlevic, M. Hajdinjak, B. Vernot, L. Skov, P. Hsieh, S. Peyregne, D. Reher, C. Hopfe, S. Nagel, T. Maricic, Q. Fu, C. Theunert, R. Rogers, P. Skoglund, M. Chintalapati, M. Dannemann, B. J. Nelson, F. M. Key, P. Rudan, Z. Kucan, I. Gusic, L. V. Golovanova, V. B. Doronichev, N. Patterson, D. Reich, E. E. Eichler, M. Slatkin, M. H. Schierup, A. M. Andres, J. Kelso, M. Meyer, S. Paabo, A high-coverage Neandertal genome from Vindija Cave in Croatia. *Science* **358**, 655–658 (2017).

13. F. Mafessoni, S. Grote, C. de Filippo, V. Slon, K. A. Kolobova, B. Viola, S. V. Markin, M. Chintalapati, S. Peyregne, L. Skov, P. Skoglund, A. I. Krivoshapkin, A. P. Derevianko, M. Meyer, J. Kelso, B. Peter, K. Prufer, S. Paabo, A high-coverage Neandertal genome from Chagyrskaya Cave. *Proc. Natl. Acad. Sci. U.S.A.* **117**, 15132–15136 (2020).

14. K. Prufer, F. Racimo, N. Patterson, F. Jay, S. Sankararaman, S. Sawyer, A. Heinze, G. Renaud, P. H. Sudmant, C. de Filippo, H. Li, S. Mallick, M. Dannemann, Q. Fu, M. Kircher, M.

Kuhlwilm, M. Lachmann, M. Meyer, M. Ongyerth, M. Siebauer, C. Theunert, A. Tandon, P. Moorjani, J. Pickrell, J. C. Mullikin, S. H. Vohr, R. E. Green, I. Hellmann, P. L. Johnson, H. Blanche, H. Cann, J. O. Kitzman, J. Shendure, E. E. Eichler, E. S. Lein, T. E. Bakken, L. V. Golovanova, V. B. Doronichev, M. V. Shunkov, A. P. Derevianko, B. Viola, M. Slatkin, D. Reich, J. Kelso, S. Paabo, The complete genome sequence of a Neanderthal from the Altai Mountains. *Nature* **505**, 43–49 (2014).

15. V. Slon, F. Mafessoni, B. Vernot, C. de Filippo, S. Grote, B. Viola, M. Hajdinjak, S. Peyregne, S. Nagel, S. Brown, K. Douka, T. Higham, M. B. Kozlikin, M. V. Shunkov, A. P. Derevianko, J. Kelso, M. Meyer, K. Prufer, S. Paabo, The genome of the offspring of a Neanderthal mother and a Denisovan father. *Nature* **561**, 113–116 (2018).

16. L. Bokelmann, M. Hajdinjak, S. Peyregne, S. Brace, E. Essel, C. de Filippo, I. Glocke, S. Grote, F. Mafessoni, S. Nagel, J. Kelso, K. Prufer, B. Vernot, I. Barnes, S. Paabo, M. Meyer, C. Stringer, A genetic analysis of the Gibraltar Neanderthals. *Proc. Natl. Acad. Sci. U.S.A.* **116**, 15610–15615 (2019).

17. M. Hajdinjak, Q. Fu, A. Hubner, M. Petr, F. Mafessoni, S. Grote, P. Skoglund, V. Narasimham, H. Rougier, I. Crevecoeur, P. Semal, M. Soressi, S. Talamo, J. J. Hublin, I. Gusic, Z. Kucan, P. Rudan, L. V. Golovanova, V. B. Doronichev, C. Posth, J. Krause, P. Korlevic, S. Nagel, B. Nickel, M. Slatkin, N. Patterson, D. Reich, K. Prufer, M. Meyer, S. Paabo, J. Kelso, Reconstructing the genetic history of late Neanderthals. *Nature* **555**, 652–656 (2018).

18. M. Meyer, J. L. Arsuaga, C. de Filippo, S. Nagel, A. Aximu-Petri, B. Nickel, I. Martinez, A. Gracia, J. M. Bermudez de Castro, E. Carbonell, B. Viola, J. Kelso, K. Prufer, S. Paabo, Nuclear DNA sequences from the Middle Pleistocene Sima de los Huesos hominins. *Nature* **531**, 504–507 (2016).

19. S. Peyregne, V. Slon, F. Mafessoni, C. de Filippo, M. Hajdinjak, S. Nagel, B. Nickel, E. Essel, A. Le Cabec, K. Wehrberger, N. J. Conard, C. J. Kind, C. Posth, J. Krause, G. Abrams, D. Bonjean, K. Di Modica, M. Toussaint, J. Kelso, M. Meyer, S. Paabo, K. Prufer, Nuclear DNA from two early Neandertals reveals 80,000 years of genetic continuity in Europe. *Sci. Adv.* **5**, eaaw5873 (2019).

20. L. Slimak, T. Vimala, A. Seguin-Orlando, L. Metz, C. Zanolli, R. Joannes-Boyau, M. Frouin, L. J. Arnold, M. Demuro, T. Deviese, D. Comeskey, M. Buckley, H. Camus, X. Muth, J. E. Lewis, H. Bocherens, P. Yvorra, C. Tenailleau, B. Duployer, H. Coqueugniot, O. Dutour, T. Higham, M. Sikora, Long genetic and social isolation in Neanderthals before their extinction. *Cell Genom.* **4**, 100593 (2024).

21. M. Meyer, M. Kircher, M. T. Gansauge, H. Li, F. Racimo, S. Mallick, J. G. Schraiber, F. Jay, K. Prufer, C. de Filippo, P. H. Sudmant, C. Alkan, Q. Fu, R. Do, N. Rohland, A. Tandon, M. Siebauer, R. E. Green, K. Bryc, A. W. Briggs, U. Stenzel, J. Dabney, J. Shendure, J. Kitzman, M. F. Hammer, M. V. Shunkov, A. P. Derevianko, N. Patterson, A. M. Andres, E. E. Eichler, M. Slatkin, D. Reich, J. Kelso, S. Paabo, A high-coverage genome sequence from an archaic Denisovan individual. *Science* **338**, 222–226 (2012).

22. 1000 Genomes Project Consortium, A. Auton, L. D. Brooks, R. M. Durbin, E. P. Garrison, H. M. Kang, J. O. Korbel, J. L. Marchini, S. McCarthy, G. A. McVean, G. R. Abecasis, A global reference for human genetic variation. *Nature* **526**, 68–74 (2015).

23. A. Bergstrom, S. A. McCarthy, R. Hui, M. A. Almarri, Q. Ayub, P. Danecek, Y. Chen, S. Felkel, P. Hallast, J. Kamm, H. Blanche, J. F. Deleuze, H. Cann, S. Mallick, D. Reich, M. S. Sandhu, P. Skoglund, A. Scally, Y. Xue, R. Durbin, C. Tyler-Smith, Insights into human genetic variation and population history from 929 diverse genomes. *Science* **367**, eaay5012 (2020).

24. R. E. Green, J. Krause, A. W. Briggs, T. Maricic, U. Stenzel, M. Kircher, N. Patterson, H. Li, W. Zhai, M. H. Fritz, N. F. Hansen, E. Y. Durand, A. S. Malaspinas, J. D. Jensen, T. Marques-Bonet, C. Alkan, K. Prufer, M. Meyer, H. A. Burbano, J. M. Good, R. Schultz, A. Aximu-Petri, A. Butthof, B. Hober, B. Hoffner, M. Siegemund, A. Weihmann, C. Nusbaum, E. S. Lander, C. Russ, N. Novod, J. Affourtit, M. Egholm, C. Verna, P. Rudan, D. Brajkovic, Z. Kucan, I. Gusic, V. B. Doronichev, L. V. Golovanova, C. Lalueza-Fox, M. de la Rasilla, J. Fortea, A. Rosas, R. W. Schmitz, P. L. F. Johnson, E. E. Eichler, D. Falush, E. Birney, J. C. Mullikin, M. Slatkin, R. Nielsen, J. Kelso, M. Lachmann, D. Reich, S. Paabo, A draft sequence of the Neandertal genome. *Science* **328**, 710–722 (2010).

25. L. N. M. Iasi, M. Chintalapati, L. Skov, A. B. Mesa, M. Hajdinjak, B. M. Peter, P. Moorjani, Neanderthal ancestry through time: Insights from genomes of ancient and present-day humans. *Science* **386**, eadq3010 (2024).

26. A. P. Sumer, H. Rougier, V. Villalba-Mouco, Y. Huang, L. N. M. Iasi, E. Essel, A. Bossoms Mesa, A. Furtwaengler, S. Peyregne, C. de Filippo, A. B. Rohrlach, F. Pierini, F. Mafessoni, H. Fewlass, E. I. Zavala, D. Mylopotamitaki, R. A. Bianco, A. Schmidt, J. Zorn, B. Nickel, A. Patova, C. Posth, G. M. Smith, K. Ruebens, V. Sinet-Mathiot, A. Stoessel, H. Dietl, J. Orschiedt, J. Kelso, H. Zeberg, K. I. Bos, F. Welker, M. Weiss, S. P. McPherron, T. Schuler, J. J. Hublin, P. Veleminsky, J. Bruzek, B. M. Peter, M. Meyer, H. Meller, H. Ringbauer, M. Hajdinjak, K. Prufer, J. Krause, Earliest modern human genomes constrain timing of Neanderthal admixture. *Nature* **638**, 711–717 (2025).

27. B. V. Halldorsson, G. Palsson, O. A. Stefansson, H. Jonsson, M. T. Hardarson, H. P. Eggertsson, B. Gunnarsson, A. Oddsson, G. H. Halldorsson, F. Zink, S. A. Gudjonsson, M. L. Frigge, G. Thorleifsson, A. Sigurdsson, S. N. Stacey, P. Sulem, G. Masson, A. Helgason, D. F. Gudbjartsson, U. Thorsteinsdottir, K. Stefansson, Characterizing mutagenic effects of recombination through a sequence-level genetic map. *Science* **363**, eaau1043 (2019).

28. R. Agren, S. Patil, X. Zhou, FinnGen, K. Sahlholm, S. Paabo, H. Zeberg, Major genetic risk factors for Dupuytren's disease are inherited from Neandertals. *Mol. Biol. Evol.* **40**, msad130 (2023).

29. E. Huerta-Sanchez, X. Jin, Asan, Z. Bianba, B. M. Peter, N. Vinckenbosch, Y. Liang, X. Yi, M. He, M. Somel, P. Ni, B. Wang, X. Ou, Huasang, J. Luosang, Z. X. Cuo, K. Li, G. Gao, Y. Yin, W. Wang, X. Zhang, X. Xu, H. Yang, Y. Li, J. Wang, J. Wang, R. Nielsen, Altitude adaptation in Tibetans caused by introgression of Denisovan-like DNA. *Nature* **512**, 194–197 (2014).

30. P. Sjodin, J. McKenna, M. Jakobsson, Estimating divergence times from DNA sequences. *Genetics* **217**, (2021).

31. S. Paabo, The human condition-a molecular approach. *Cell* **157**, 216–226 (2014).

32. M. H. Meisler, C. Reinke, A sensitive fluorescent assay for N-acetyltransferase activity in human lymphocytes from newborns and adults. *Clin. Chim. Acta* **96**, 91–96 (1979).

33. T. Imai, K. Tanaka, T. Yonemitsu, Y. Yakushiji, K. Ohura, Elucidation of the intestinal absorption of para-aminobenzoic acid, a marker for dietary intake. *J. Pharm. Sci.* **106**, 2881–2888 (2017).

34. E. W. Sutherland Jr, W. D. Wosilait, Inactivation and activation of liver phosphorylase. *Nature* **175**, 169–170 (1955).

35. E. K. Keenan, D. K. Zachman, M. D. Hirschey, Discovering the landscape of protein modifications. *Mol. Cell* **81**, 1868–1878 (2021).

36. P. Van Damme, M. Lasa, B. Polevoda, C. Gazquez, A. Elosegui-Artola, D. S. Kim, E. De Juan-Pardo, K. Demeyer, K. Hole, E. Larrea, E. Timmerman, J. Prieto, T. Arnesen, F. Sherman, K. Gevaert, R. Aldabe, N-terminal acetylome analyses and functional insights of the N-terminal acetyltransferase NatB. *Proc. Natl. Acad. Sci. U.S.A.* **109**, 12449–12454 (2012).

37. Z. Chen, P. A. Cole, Synthetic approaches to protein phosphorylation. *Curr. Opin. Chem. Biol.* **28**, 115–122 (2015).

38. Z. Tang, C. Li, B. Kang, G. Gao, C. Li, Z. Zhang, GEPIA: A web server for cancer and normal gene expression profiling and interactive analyses. *Nucleic Acids Res.* **45**, W98-W102 (2017).

39. P. Mertins, D. R. Mani, K. V. Ruggles, M. A. Gillette, K. R. Clauser, P. Wang, X. Wang, J. W. Qiao, S. Cao, F. Petralia, E. Kawaler, F. Mundt, K. Krug, Z. Tu, J. T. Lei, M. L. Gatza, M. Wilkerson, C. M. Perou, V. Yellapantula, K. L. Huang, C. Lin, M. D. McLellan, P. Yan, S. R. Davies, R. R. Townsend, S. J. Skates, J. Wang, B. Zhang, C. R. Kinsinger, M. Mesri, H. Rodriguez, L. Ding, A. G. Paulovich, D. Fenyo, M. J. Ellis, S. A. Carr, C. Nci, Proteogenomics connects somatic mutations to signalling in breast cancer. *Nature* **534**, 55–62 (2016).

40. K. L. Huang, S. Li, P. Mertins, S. Cao, H. P. Gunawardena, K. V. Ruggles, D. R. Mani, K. R. Clauser, M. Tanioka, J. Usary, S. M. Kavuri, L. Xie, C. Yoon, J. W. Qiao, J. Wrobel, M. A. Wyczalkowski, P. Erdmann-Gilmore, J. E. Snider, J. Hoog, P. Singh, B. Niu, Z. Guo, S. Q. Sun,

S. Sanati, E. Kawaler, X. Wang, A. Scott, K. Ye, M. D. McLellan, M. C. Wendl, A. Malovannaya, J. M. Held, M. A. Gillette, D. Fenyo, C. R. Kinsinger, M. Mesri, H. Rodriguez, S. R. Davies, C. M. Perou, C. Ma, R. Reid Townsend, X. Chen, S. A. Carr, M. J. Ellis, L. Ding, Proteogenomic integration reveals therapeutic targets in breast cancer xenografts. *Nat. Commun.* **8**, 14864 (2017).

41. M. A. Gillette, S. Satpathy, S. Cao, S. M. Dhanasekaran, S. V. Vasaikar, K. Krug, F. Petralia, Y. Li, W. W. Liang, B. Reva, A. Krek, J. Ji, X. Song, W. Liu, R. Hong, L. Yao, L. Blumenberg, S. R. Savage, M. C. Wendl, B. Wen, K. Li, L. C. Tang, M. A. MacMullan, S. C. Avanessian, M. H. Kane, C. J. Newton, M. Cornwell, R. B. Kothadia, W. Ma, S. Yoo, R. Mannan, P. Vats, C. Kumar-Sinha, E. A. Kawaler, T. Omelchenko, A. Colaprico, Y. Geffen, Y. E. Maruvka, F. da Veiga Leprevost, M. Wiznerowicz, Z. H. Gumus, R. R. Veluswamy, G. Hostetter, D. I. Heiman, M. A. Wyczalkowski, T. Hiltke, M. Mesri, C. R. Kinsinger, E. S. Boja, G. S. Omenn, A. M. Chinnaiyan, H. Rodriguez, Q. K. Li, S. D. Jewell, M. Thiagarajan, G. Getz, B. Zhang, D. Fenyo, K. V. Ruggles, M. P. Cieslik, A. I. Robles, K. R. Clauser, R. Govindan, P. Wang, A. I. Nesvizhskii, L. Ding, D. R. Mani, S. A. Carr, Clinical Proteomic Tumor Analysis Consortium, Proteogenomic characterization reveals therapeutic vulnerabilities in lung adenocarcinoma. *Cell* **182**, 200–225.e35 (2020).

42. S. Vasaikar, C. Huang, X. Wang, V. A. Petyuk, S. R. Savage, B. Wen, Y. Dou, Y. Zhang, Z. Shi, O. A. Arshad, M. A. Gritsenko, L. J. Zimmerman, J. E. McDermott, T. R. Clauss, R. J. Moore, R. Zhao, M. E. Monroe, Y. T. Wang, M. C. Chambers, R. J. C. Slebos, K. S. Lau, Q. Mo, L. Ding, M. Ellis, M. Thiagarajan, C. R. Kinsinger, H. Rodriguez, R. D. Smith, K. D. Rodland, D. C. Liebler, T. Liu, B. Zhang, Clinical Proteomic Tumor Analysis Consortium, Proteogenomic analysis of human colon cancer reveals new therapeutic opportunities. *Cell* **177**, 1035–1049.e19 (2019).

43. J. Leger, M. Kempf, G. Lee, R. Brandt, Conversion of serine to aspartate imitates phosphorylation-induced changes in the structure and function of microtubule-associated protein tau. *J. Biol. Chem.* **272**, 8441–8446 (1997).

44. N. Dissmeyer, A. Schnittger, *Plant Kinases: Methods and Protocols* (Humana Press, 2011).

45. H. Wu, L. Dombrovsky, W. Tempel, F. Martin, P. Loppnau, G. H. Goodfellow, D. M. Grant, A. N. Plotnikov, Structural basis of substrate-binding specificity of human arylamine *N*-acetyltransferases. *J. Biol. Chem.* **282**, 30189–30197 (2007).

46. J. Abramson, J. Adler, J. Dunger, R. Evans, T. Green, A. Pritzel, O. Ronneberger, L. Willmore, A. J. Ballard, J. Bambrick, S. W. Bodenstein, D. A. Evans, C. C. Hung, M. O'Neill, D. Reiman, K. Tunyasuvunakool, Z. Wu, A. Zemgulyte, E. Arvaniti, C. Beattie, O. Bertolli, A. Bridgland, A. Cherepanov, M. Congreve, A. I. Cowen-Rivers, A. Cowie, M. Figurnov, F. B. Fuchs, H. Gladman, R. Jain, Y. A. Khan, C. M. R. Low, K. Perlin, A. Potapenko, P. Savy, S. Singh, A. Stecula, A. Thillaisundaram, C. Tong, S. Yakneen, E. D. Zhong, M. Zielinski, A. Zidek, V. Bapst, P. Kohli, M. Jaderberg, D. Hassabis, J. M. Jumper, Accurate structure prediction of biomolecular interactions with AlphaFold 3. *Nature* **630**, 493–500 (2024).

47. H. X. Zhou, X. Pang, Electrostatic interactions in protein structure, folding, binding, and condensation. *Chem. Rev.* **118**, 1691–1741 (2018).

48. V. A. Smelt, A. Upton, J. Adjaye, M. A. Payton, S. Boukouvala, N. Johnson, H. J. Mardon, E. Sim, Expression of arylamine *N*-acetyltransferases in pre-term placentas and in human pre-implantation embryos. *Hum. Mol. Genet.* **9**, 1101–1107 (2000).

49. D. W. Hein, M. A. Doll, A. J. Fretland, M. A. Leff, S. J. Webb, G. H. Xiao, U. S. Devanaboyina, N. A. Nangju, Y. Feng, Molecular genetics and epidemiology of the NAT1 and NAT2 acetylation polymorphisms. *Cancer Epidemiol. Biomarkers Prev.* **9**, 29–42 (2000).

50. D. W. Hein, Molecular genetics and function of NAT1 and NAT2: Role in aromatic amine metabolism and carcinogenesis. *Mutat. Res.* **506-507**, 65–77 (2002).

51. G. Perez-Mejias, A. Velazquez-Cruz, A. Guerra-Castellano, B. Banos-Jaime, A. Diaz-Quintana, K. Gonzalez-Arzola, M. Angel De la Rosa, I. Diaz-Moreno, Exploring protein phosphorylation by combining computational approaches and biochemical methods. *Comput. Struct. Biotechnol. J.* **18**, 1852–1863 (2020).

52. L. A. Stanley, A. J. Copp, J. Pope, S. Rolls, V. Smelt, V. H. Perry, E. Sim, Immunochemical detection of arylamine N-acetyltransferase during mouse embryonic development and in adult mouse brain. *Teratology* **58**, 174–182 (1998).

53. A. Ward, M. J. Summers, E. Sim, Purification of recombinant human N-acetyltransferase type 1 (NAT1) expressed in E. coli and characterization of its potential role in folate metabolism. *Biochem. Pharmacol.* **49**, 1759–1767 (1995).

54. W. Cao, D. Strnatka, C. A. McQueen, R. J. Hunter, R. P. Erickson, N-acetyltransferase 2 activity and folate levels. *Life Sci.* **86**, 103–106 (2010).

55. A. Ward, D. Hickman, J. W. Gordon, E. Sim, Arylamine N-acetyltransferase in human red blood cells. *Biochem. Pharmacol.* **44**, 1099–1104 (1992).

56. K. S. Crider, Y. P. Qi, L. F. Yeung, C. T. Mai, L. Head Zauche, A. Wang, K. Daniels, J. L. Williams, Folic acid and the prevention of birth defects: 30 Years of opportunity and controversies. *Annu. Rev. Nutr.* **42**, 423–452 (2022).

57. S. L. Carmichael, G. M. Shaw, W. Yang, D. M. Iovannisci, E. Lammer, Risk of limb deficiency defects associated with *NAT1*, *NAT2*, *GSTT1*, *GSTM1*, and *NOS3* genetic variants, maternal smoking, and vitamin supplement intake. *Am. J. Med. Genet. A* **140**, 1915–1922 (2006).

58. E. J. Lammer, G. M. Shaw, D. M. Iovannisci, R. H. Finnell, Periconceptional multivitamin intake during early pregnancy, genetic variation of acetyl-*N*-transferase 1 (*NAT1*), and risk for orofacial clefts. *Birth Defects Res. A Clin. Mol. Teratol.* **70**, 846–852 (2004).

59. E. J. Lammer, G. M. Shaw, D. M. Iovannisci, J. Van Waes, R. H. Finnell, Maternal smoking and the risk of orofacial clefts: Susceptibility with NAT1 and NAT2 polymorphisms. *Epidemiology* **15**, 150–156 (2004).

60. F. Tajima, Statistical method for testing the neutral mutation hypothesis by DNA polymorphism. *Genetics* **123**, 585–595 (1989).

61. A. C. Ross, B. H. Caballero, R. J. Cousins, K. L. Tucker, T. R. Ziegler, Eds., *Modern Nutrition in Health and Disease: Eleventh Edition* [Wolters Kluwer Health Adis (ESP), 2012].

62. D. J. McKillop, K. Pentieva, D. Daly, J. M. McPartlin, J. Hughes, J. J. Strain, J. M. Scott, H. McNulty, The effect of different cooking methods on folate retention in various foods that are amongst the major contributors to folate intake in the UK diet. *Br. J. Nutr.* **88**, 681–688 (2002).

63. D. Wang, M. F. Para, S. L. Koletar, W. Sadee, Human N-acetyltransferase 1 *10 and *11 alleles increase protein expression through distinct mechanisms and associate with sulfamethoxazole-induced hypersensitivity. *Pharmacogenet. Genomics* **21**, 652–664 (2011).

64. M. J. Machiela, S. J. Chanock, LDlink: A web-based application for exploring population-specific haplotype structure and linking correlated alleles of possible functional variants. *Bioinformatics* **31**, 3555–3557 (2015).

65. A. Kong, G. Thorleifsson, D. F. Gudbjartsson, G. Masson, A. Sigurdsson, A. Jonasdottir, G. B. Walters, A. Jonasdottir, A. Gylfason, K. T. Kristinsson, S. A. Gudjonsson, M. L. Frigge, A. Helgason, U. Thorsteinsdottir, K. Stefansson, Fine-scale recombination rate differences between sexes, populations and individuals. *Nature* **467**, 1099–1103 (2010).

66. L. Skov, R. Hui, V. Shchur, A. Hobolth, A. Scally, M. H. Schierup, R. Durbin, Detecting archaic introgression using an unadmixed outgroup. *PLOS Genet.* **14**, e1007641 (2018).

67. M. Byrska-Bishop, U. S. Evani, X. Zhao, A. O. Basile, H. J. Abel, A. A. Regier, A. Corvelo, W. E. Clarke, R. Musunuri, K. Nagulapalli, S. Fairley, A. Runnels, L. Winterkorn, E. Lowy, Human Genome Structural Variation Consortium, P. Flicek, S. Germer, H. Brand, I. M. Hall, M. E. Talkowski, G. Narzisi, M. C. Zody, High-coverage whole-genome sequencing of the expanded 1000 Genomes Project cohort including 602 trios. *Cell* **185**, 3426–3440.e19 (2022).

68. M. M. Bradford, A rapid and sensitive method for the quantitation of microgram quantities of protein utilizing the principle of protein-dye binding. *Anal. Biochem.* **72**, 248–254 (1976).

69. N. Blom, T. Sicheritz-Ponten, R. Gupta, S. Gammeltoft, S. Brunak, Prediction of post-translational glycosylation and phosphorylation of proteins from the amino acid sequence. *Proteomics* **4**, 1633–1649 (2004).

70. R. Apweiler, A. Bairoch, C. H. Wu, W. C. Barker, B. Boeckmann, S. Ferro, E. Gasteiger, H. Huang, R. Lopez, M. Magrane, M. J. Martin, D. A. Natale, C. O'Donovan, N. Redaschi, L. S. Yeh, UniProt: The Universal Protein knowledgebase. *Nucleic Acids Res.* **32**, D115–D 119 (2004).

71. P. V. Hornbeck, B. Zhang, B. Murray, J. M. Kornhauser, V. Latham, E. Skrzypek, PhosphoSitePlus, 2014: Mutations, PTMs and recalibrations. *Nucleic Acids Res.* **43**, D512–D520 (2015).

72. C.-R. Chung, Y. Tang, Y.-P. Chiu, S. Li, W.-K. Hsieh, L. Yao, Y.-C. Chiang, Y. Pang, G.-T. Chen, K.-C. Chou, Y. S. Paik, P. L. Tran, C.-P. Lin, Y.-M. Kao, Y.-J. Chen, W.-C. Chang, J. B.-K. Hsu, J.-T. Horng, T.-Y. Lee, dbPTM 2025 update: comprehensive integration of PTMs and proteomic data for advanced insights into cancer research. *Nucleic Acids Res.* **53**, D377-D386 (2025).

73. Q. Dong, D. Shen, J. Ye, J. Chen, J. Li, PhosCancer: A comprehensive database for investigating protein phosphorylation in human cancer. *iScience* **27**, 111060 (2024).

74. F. Desiere, E. W. Deutsch, N. L. King, A. I. Nesvizhskii, P. Mallick, J. Eng, S. Chen, J. Eddes, S. N. Loevenich, R. Aebersold, The PeptideAtlas project. *Nucleic Acids Res.* **34**, D655–658 (2006).

75. E. C. Meng, T. D. Goddard, E. F. Pettersen, G. S. Couch, Z. J. Pearson, J. H. Morris, T. E. Ferrin, UCSF ChimeraX: Tools for structure building and analysis. *Protein Sci.* **32**, e4792 (2023).

76. W. Tian, C. Chen, X. Lei, J. Zhao, J. Liang, CASTp 3.0: Computed atlas of surface topography of proteins. *Nucleic Acids Res.* **46**, W363–W367 (2018).

77. B. Yariv, E. Yariv, A. Kessel, G. Masrati, A. B. Chorin, E. Martz, I. Mayrose, T. Pupko, N. Ben-Tal, Using evolutionary data to make sense of macromolecules with a “face-lifted” ConSurf. *Protein Sci.* **32**, e4582 (2023).

78. F. Madeira, N. Madhusoodanan, J. Lee, A. Eusebi, A. Niewielska, A. R. N. Tivey, R. Lopez, S. Butcher, The EMBL-EBI Job Dispatcher sequence analysis tools framework in 2024. *Nucleic Acids Res.* **52**, W521–W525 (2024).

79. N. C. Hughes, S. A. Janezic, K. L. McQueen, M. A. Jewett, T. Castranio, D. A. Bell, D. M. Grant, Identification and characterization of variant alleles of human acetyltransferase NAT1 with defective function using p-aminosalicylate as an in-vivo and in-vitro probe. *Pharmacogenetics* **8**, 55–66 (1998).

80. J. H. de Leon, K. P. Vatsis, W. W. Weber, Characterization of naturally occurring and recombinant human N-acetyltransferase variants encoded by NAT1. *Mol. Pharmacol.* **58**, 288–299 (2000).

81. S. A. Vaziri, N. C. Hughes, H. Sampson, G. Darlington, M. A. Jewett, D. M. Grant, Variation in enzymes of arylamine procarcinogen biotransformation among bladder cancer patients and control subjects. *Pharmacogenetics* **11**, 7–20 (2001).

82. A. J. Fretland, M. A. Doll, M. A. Leff, D. W. Hein, Functional characterization of nucleotide polymorphisms in the coding region of N-acetyltransferase 1. *Pharmacogenetics* **11**, 511–520 (2001).

83. A. J. Fretland, M. A. Doll, Y. Zhu, L. Smith, M. A. Leff, D. W. Hein, Effect of nucleotide substitutions in N-acetyltransferase-1 on N-acetylation (deactivation) and O-acetylation (activation) of arylamine carcinogens: implications for cancer predisposition. *Cancer Detect. Prev.* **26**, 10–14 (2002).

84. M. A. Doll, W. Jiang, A. C. Deitz, T. D. Rustan, D. W. Hein, Identification of a novel allele at the human NAT1 acetyltransferase locus. *Biochem. Biophys. Res. Commun.* **233**, 584–591 (1997).

85. Y. Zhu, D. W. Hein, Functional effects of single nucleotide polymorphisms in the coding region of human *N*-acetyltransferase 1. *Pharmacogenomics J.* **8**, 339–348 (2008).

86. A. Risch, V. Smelt, D. Lane, L. Stanley, W. van der Slot, A. Ward, E. Sim, Arylamine N-acetyltransferase in erythrocytes of cystic fibrosis patients. *Pharmacol. Toxicol.* **78**, 235–240 (1996).

87. M. A. Payton, E. Sim, Genotyping human arylamine N-acetyltransferase type 1 (NAT1): The identification of two novel allelic variants. *Biochem. Pharmacol.* **55**, 361–366 (1998).

88. C. Bruhn, J. Brockmoller, I. Cascorbi, I. Roots, H. H. Borchert, Correlation between genotype and phenotype of the human arylamine N-acetyltransferase type 1 (NAT1). *Biochem. Pharmacol.* **58**, 1759–1764 (1999).

89. X. Zhangwei, X. Jianming, M. Qiao, X. Xinhua, N-Acetyltransferase-1 gene polymorphisms and correlation between genotype and its activity in a central Chinese Han population. *Clin. Chim. Acta* **371**, 85–91 (2006).

90. L. Chen, A. B. Wolf, W. Fu, L. Li, J. M. Akey, Identifying and interpreting apparent Neanderthal ancestry in African individuals. *Cell* **180**, 677–687.e16 (2020).

91. M. Cardoso-Moreira, J. Halbert, D. Valloton, B. Velten, C. Chen, Y. Shao, A. Liechti, K. Ascencao, C. Rummel, S. Ovchinnikova, P. V. Mazin, I. Xenarios, K. Harshman, M. Mort, D. N. Cooper, C. Sandi, M. J. Soares, P. G. Ferreira, S. Afonso, M. Carneiro, J. M. A. Turner, J. L. VandeBerg, A. Fallahshahroudi, P. Jensen, R. Behr, S. Lisgo, S. Lindsay, P. Khaitovich, W. Huber, J. Baker, S. Anders, Y. E. Zhang, H. Kaessmann, Gene expression across mammalian organ development. *Nature* **571**, 505–509 (2019).
